# Shedding Light on the Photophysics and Photochemistry of I-Motifs Using Quantum Mechanical Calculations

**DOI:** 10.3390/ijms241612614

**Published:** 2023-08-09

**Authors:** Roberto Improta

**Affiliations:** Consiglio Nazionale delle Ricerche, Istituto di Biostrutture e Bioimmagini (IBB-CNR), Via De Amicis 95, I-80145 Napoli, Italy; roberto.improta@cnr.it

**Keywords:** protonated cytosine, non-canonical DNA structure, TD-DFT

## Abstract

I-motifs are non-canonical DNA structures formed by intercalated hemiprotonated (CH·C)${}^{+}$ pairs, i.e., formed by a cytosine (C) and a protonated cytosine (CH${}^{+}$), which are currently drawing great attention due to their biological relevance and promising nanotechnological properties. It is important to characterize the processes occurring in I-motifs following irradiation by UV light because they can lead to harmful consequences for genetic code and because optical spectroscopies are the most-used tools to characterize I-motifs. By using time-dependent DFT calculations, we here provide the first comprehensive picture of the photoactivated behavior of the (CH·C)${}^{+}$ core of I-motifs, from absorption to emission, while also considering the possible photochemical reactions. We reproduce and assign their spectral signatures, i.e., infrared, absorption, fluorescence and circular dichroism spectra, disentangling the underlying chemical–physical effects. We show that the main photophysical paths involve C and CH${}^{+}$ bases on adjacent steps and, using this basis, interpret the available time-resolved spectra. We propose that a photodimerization reaction can occur on an excited state with strong C→CH${}^{+}$ charge transfer character and examine some of the possible photoproducts. Based on the results reported, some future perspectives for the study of I-motifs are discussed.

## 1. Introduction

**Figure 1:**
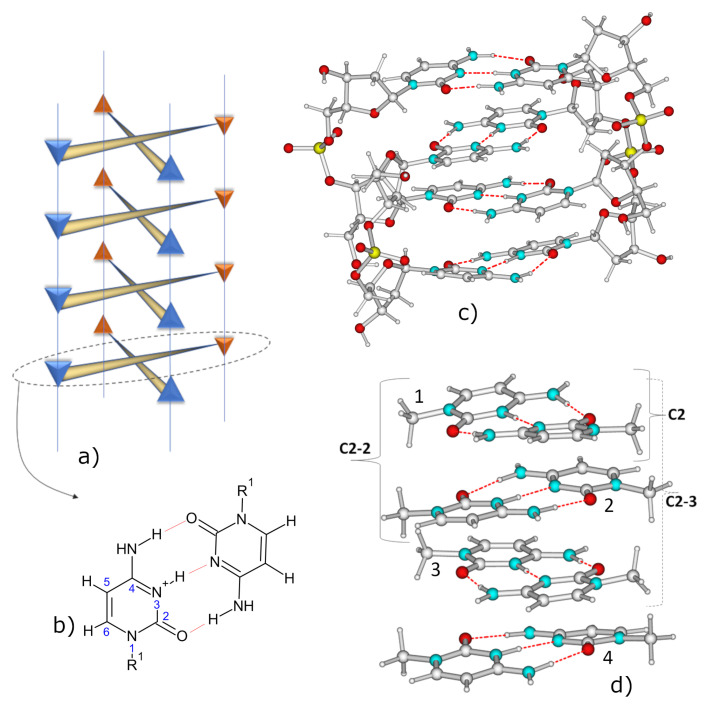
Schematic drawing of (**a**) an I motif structure; blue and yellow triangles represent CH${}^{+}$ and C bases, respectively; (**b**) (CH·C)${}^{+}$ dimer; (**c**) dC${}_{2}$ 4 model; (**d**) C2-4 models, with C2, C2-2, C2-3. Color code: C (grey), H (white), O (red), N (light blue), P (yellow).

DNA and RNA regions rich in cytosine (C) can fold in a peculiar ’intercalated’ structure, commonly referred to as an I-motif [1,2,3,4], stabilized by hemiprotonated (CH·C)${}^{+}$ pairs (see Figure 1), i.e., two base-paired cytidines, ’sharing’ one proton between their N3 atoms. These structures, which can be formed even at higher pH than the cytosine pK${}_{a}$(∼ 4.2 [5]), have recently been shown to also be present at physiological pH [6,7] and to also exist in vivo [8,9]—in regulatory regions of the human genome [9]. I-motifs have, thus, drawn a lot of attention for their involvement in cellular processes [10,11,12,13,14,15,16] and, consequently, as targets for medical applications [17,18,19]. Moreover, they show promising potential in the fields of nanotechnology, biosensing and bioanalytics, which exploit their dependence on the pH of the embedding medium [20,21,22,23,24,25,26,27]. The most recent studies enabled significant advances in our knowledge of key issues concerning I-motifs, such as, inter alia, their folding mechanism and the factors affecting their stability, such as the number of possible (CH·C)${}^{+}$ pairs and the nature of the bases in the loops [28,29,30,31,32,33,34,35,36,37,38].

On the other hand, also due to their dynamic structural behavior, many basic features of I-motifs are still poorly understood, hampering full exploitation of the potential of these structures. This study focuses on the processes triggered in I-motifs by UV absorption, a field not yet investigated in detail but whose elucidation would be extremely useful. Absorption and electronic circular dichroism spectra are indeed the most basic tools to identify the presence of I-motif structures and monitor their static and dynamic features [34,39,40]. On the other hand, these techniques do not give direct access to the structural details at the atomic level, which would be instead extremely valuable, considering that the number of I-motif experimental tri-dimensional structures is still limited [1,4,39]. The first goal of this study was, thus, a full assignment of the peculiar absorption and circular dichroism spectra of I-motifs, identifying the involved excited states and the most important effects modulating their spectral shapes. Then, we focused on the excited state processes occurring in I-motifs, due to their potential biological (and technological) relevance.

UV absorption can indeed trigger a cascade of oxidative processes in nucleic acids (NA) which can have harmful consequences for living beings [41,42,43,44]. As a consequence, in the last decades, the study of the photoactivated dynamics of NA has been an extremely lively research field, and huge experimental and computational efforts—too many to be exhaustively reviewed here—have been devoted to identifying and characterizing the processes, either enabling the NAs to dissipate (as heat) the energy deposited by UV irradiation or, on the contrary, leading to oxidative damage [41,45,46,47,48,49,50,51]. These studies have enabled important advances in our understanding of the excited state behavior in DNA duplexes [41,45] and also in non-canonical structures such as guanine quadruplexes [52,53,54].

No general picture is available for the excited state process occurring on I-motifs, although some interesting time-resolved (TR) studies have highlighted their very rich photophysics and photochemistry [39,55,56,57,58,59].

There are several reasons why the excited electronic states of cytosine-rich DNA sequences, such as those forming I-motifs, could be involved in pathological processes. First, the absorption spectrum of I-motifs is significantly red-shifted with respect to that of duplexes [39], and they also absorb at frequencies greater than 300 nm, where protection from atmospheric ozone is smaller and the solar spectrum at Earth’s surface is more intense. Second, photochemical products [57] and excited states existing at the nanosecond time-scale have been shown to be present in I-motifs [55,56], increasing the possibility that ’secondary’ radical reactions occur. More generally, photoactivated cytosine oxidative processes have become particularly important [41,42,60,61], since cytosine deamination and, therefore, C→U mutation and GC→AT transversion can be carcinogenetic [62].

In this study, we thus tried to disentangle the most important photophysical and photochemical processes occurring in I-motifs constituted on a certain number of (CH·C)${}^{+}$ pairs (see Figure 1) by focusing on their ’core’, while also considering the phosphodeoxyrobose backbone. We made use of quantum mechanical calculations, using a similar approach to that profitably adopted in our previous studies on the photoactivated dynamics in DNA [45,51]. In the last decade, quantum mechanical calculations and, more generally, computational techniques have indeed proven to be an extremely important, if not fundamental, complement to experimental approaches to advance our understanding, at the molecular level, of the oxidative processes in NAs [50,51].

## 2. Results

### 2.1. IR Spectra

As a first step, we located the minima of our I-motif models in the ground electronic state (GS). The optimized structures exhibited the typical features present in the experimental X-ray or NMR structures of the ’inner’ core of I-motifs [4,39], with an inter-step distance of 3.1∼3.2 Å and the axis of successive (CH·C)${}^{+}$ dimers being almost orthogonal. There were many close sugar–sugar contacts, which have been indeed shown to be critical for the stability of I-motifs [4,63]. In particular, we observed strong C1′-H⋯O4′ interactions (bond distances 2.3∼2.4 Å) between the sugars of neighboring bases, in agreement with experimental indications [64].

**Figure 2:**
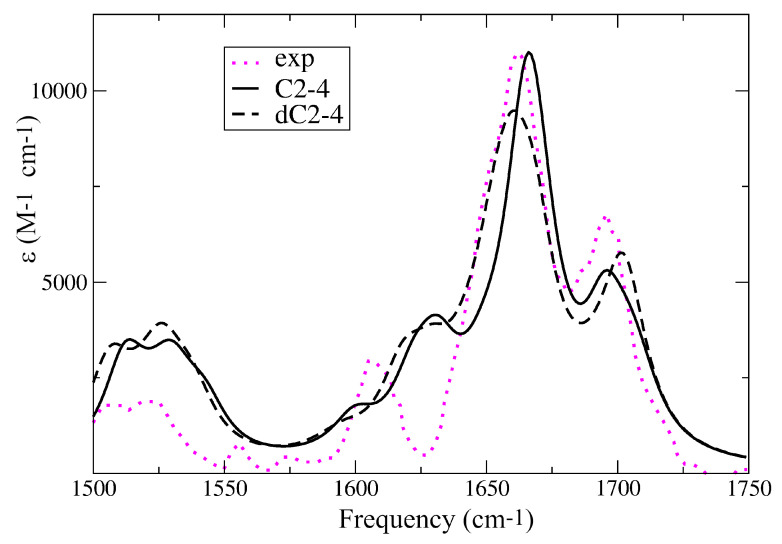
Computed IR spectra for C2-4 (black line) and dC2-4 (black dashed lines). PCM/M052X/6-31G(d) calculations. The experimental spectrum measured for (dC)${}_{30}$ at pH 5.5 [55] is also reported (dotted magenta line).

I-motifs are characterized by distinctive IR spectra, quite different from those typical of single and double strands [55,57,65]. As shown in Figure 2, in the fingerprint 1450–1750 cm${}^{-1}$ region, the experimental IR spectrum of an I-motif (in this case formed by (dC)${}_{30}$ at pH 5.5) [55] exhibits a strong peak at ∼1670 cm${}^{-1}$, with a shoulder at ∼1650 cm${}^{-1}$ and another peak (∼50 % less intense) at ∼1700 cm${}^{-1}$. Two less intense and broad peaks are then found at ∼1610 cm${}^{-1}$ and in the 1500∼1520 cm${}^{-1}$ region. These features were well reproduced by our computed spectra, concerning the position and the relative intensity of the different peaks. As shown in the SI (Section S2, Figures S1 and S2), we also correctly described the effect of N3 protonation on I-motif-like structure. Though the different vibrational modes were strongly mixed, we could assign the peak at ∼1700 cm${}^{-1}$ to the combination of the C′O′ stretching modes in CH${}^{+}$ bases. Then, the most intense peak at ∼1670 cm${}^{-1}$ derived from the combination of the ring stretching modes of both C and CH${}^{+}$ bases, which were strongly coupled by the multiple hydrogen-bond and stacking interactions. The less intense feature at 1625 cm${}^{-1}$, likely corresponding to the one appearing at 1610 cm${}^{-1}$ in the experiments, was associated with the CO stretching in the C bases. Finally, ring stretches of C and CH${}^{+}$ were also responsible for the broad band above 1500 cm${}^{-1}$, whose intensity decreased upon N3 protonation (see Figure S2 in the Supplemental Information (SI)) [55].

The IR spectra computed for C2-4, C2-3, C2-2 and C2 are reported in Figure S3 in the SI. They were pretty similar to those obtained including the backbone, and showed that the high-energy ∼1700 cm${}^{-1}$ peak slightly red-shifted when the number of (CH·C)${}^{+}$ pairs increased, improving the agreement with the experiments.

### 2.2. Assignment of the Steady State Optical Spectra

**Figure 3:**
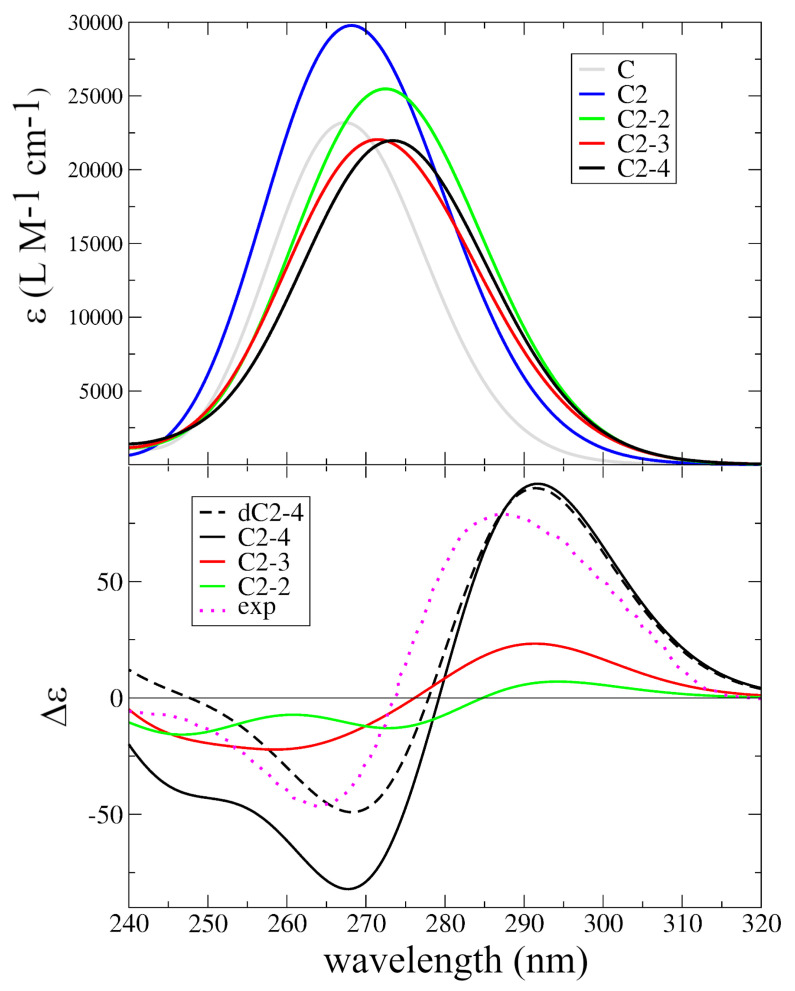
Absorption (top) and ECD (bottom) spectra computed for the different I-motif models. TD-PCM/M052X/6-31G(d) transition shifted by −0.6 eV and broadened with a Gaussian with HWHM = 0.2 eV. The intensities of absorption spectra are normalized with respect to the number of C2 pairs. Δϵ expressed in 10${}^{-40}$ esu${}^{2}$ cm${}^{2}$. The experimental spectrum (in mdeg) measured for (dC)${}_{30}$ at pH 5.5 [55] is also reported (dotted magenta line).

In Figure 3, we report the absorption spectra computed for the different I-motif models, compared with that computed in water for C at the same level of theory. It is well known that the formation of an I-motif at low pH is mirrored by some characteristic changes in the absorption spectra [39,56,58]. When compared to a mixture of the constituents or to the same sequences at pH 7, the absorption spectrum exhibits a weak red-shift of the maximum and a significant increase in the absorption in the red-wing (in the range 280∼320 nm) [39,56,58]. As shown in Figure 3, our calculations fully captured the experimental trends. Indeed, the maxima of the absorption in the I-motifs’ structure (C2-2, C2-3, C2-4), at ∼275 nm, were weakly red-shifted with respect to that for C. More importantly, the absorption intensity above 280 nm significantly increased. Interestingly, our calculations predicted that the formation of the (CH·C)${}^{+}$ pair leads to a small increase in the intensity, whereas the formation of the I-motif is mirrored by a decrease in the intensity, with the typical hypochromic effect of DNA duplex formation. Figure S4 in the SI shows that the inclusion of the phosphodeoxyribose backbone led to a moderate increase in the intensity and to a weak red-shift in the absorption maximum.

As shown in Figure 3 bottom, the computed ECD spectra were also in very good agreement with the experimental ones. The ECD spectra of I-motifs also have a typical shape, with a strong positive band peaking at ∼285 nm and another positive one at ∼265 nm [39,56,58,66]. The ECD spectra computed for the most representative models, dC2-4 and C2-4, indeed exhibited a strong positive peak at ∼290 nm and a negative one at ∼270 nm, and the relative intensity of the two peaks was almost quantitatively reproduced by the calculations on dC2-4. Confirming the experimental indications [34,40], the intensity of the two peaks increased with the number of (CH·C)${}^{+}$ pairs. The C2-3 spectrum also had the correct shapes, but the computed intensity was too weak and the separation between the positive and negative peaks was too large. Finally, the The C2-2 spectrum exhibited a small increase in the optical activity at 290 nm, but its shape was not very similar to that typical of I-motifs. These results strongly support the reliability of our computational approach and provide important indications about the interactions responsible for the experimental features. Indeed, it is clear that the stacking between non-proximal (CH·C)${}^{+}$ pairs, e.g., between the pairs labeled 1/3 and 2/4 in Figure 1, regulates the ECD shape and intensity. This is likely due to the fact that the stacking between the adjacent pair is not large, whereas the 1/3 and 2/4 base rings, though quite distant, are almost perfectly overlapped.

We checked that ECD spectra computed with CAM-B3LYP and the ωB97XD functionals, or with the larger 6-311+G(d,p) basis set, were similar to those described above (see Figure S5 in the Supplemental Information (SI)), supporting the reliability of our computational analysis. Finally, as shown in Figure S6 in the Supplemental Information (SI), the computed shape did not depend on the choice of a particular hemiprotonation pattern for dC2-4.

After assessing their accuracy, we could use our calculation to assign the spectra, i.e., identify the states responsible for the different features. To this aim, the excited states of (CH·C)${}^{+}$ pair provided a useful reference (see Figure S8 in the SI). As discussed in detail in ref. [67], the lowest-energy bright excited state in (CH·C)${}^{+}$ is a $\pi \pi^{*}$ transition localized on the CH${}^{+}$ base, mostly involving the C5=C6 double bond. In the following, we label this kind of excited state as CH${}^{+}$-$\pi \pi^{*}$. CH${}^{+}$-$\pi \pi^{*}$ is slightly (∼0.15 eV) more stable than an analogous transition, localized on the C base (hereafter, C-$\pi \pi^{*}$), which is very similar to the lowest-energy excited state of a ‘free’ C base [68].

In Section S2.4.1 of the Supplemental Information (SI), the interested reader can find a more detailed description of the excited states of C2-2 (see Figure S9), showing how the formation of the I-motif, and the consequent coupling between the stacked bases, induces the coupling between the CH${}^{+}$-$\pi \pi^{*}$, on the one hand, and between the C-$\pi \pi^{*}$ transitions, on the other hand. Moreover, CT states involving two stacked bases are possible. The most stable ones, ∼0.3 eV less stable than C-$\pi \pi^{*}$ transitions, involve the transfer of an electron from a C towards the stacked CH${}^{+}$, which we label as C-CH${}^{+}$-CT states.

**Figure 4:**
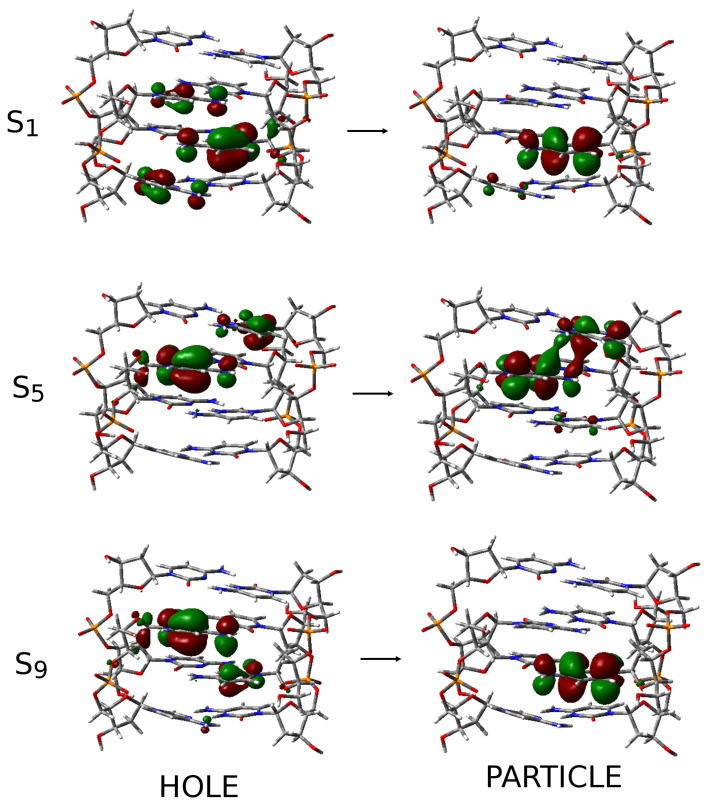
Schematic description of three excited states representative of the main excited states families in dC2-4. The excited state can be represented by an electron transferred from the NTO on the left (HOLE) to that on the right (PARTICLE).

These excited states are the main players involved in the lowest-energy absorption band of I-motifs. In longer sequences, we find a group of excited states deriving from the mixing of the CH${}^{+}$-$\pi \pi^{*}$ transitions, slightly red-shifted with respect to those deriving from C-$\pi \pi^{*}$ transitions. In Figure 4, we show the two most stable excited states of the CH${}^{+}$-$\pi \pi^{*}$ (S${}_{1}$) and C-$\pi \pi^{*}$ (S${}_{5}$) families for dC2-4. Then, we found the C→CH${}^{+}$ CT states (see, for example, S${}_{9}$ in Figure 4). It is important to remind the reader that these assignments mainly capture the ’dominant’ character of the excited states, which are always mixed, which also depend on the conformational fluctuations in the chain. For example, inspection of Figure 4 shows that S${}_{1}$ has a small C→CH${}^{+}$ CT character. In the SI, we show how these excited states contribute to the typical ECD signature of I-motifs (Figure S10).

### 2.3. Excited State Minima

As the next step, we optimized the geometry of the lowest-energy excited states for the most representative models: the minimum one, i.e., C2-2 (for which we performed the most thorough analysis), and the largest ones, i.e., C2-4 and dC2-4.

**Figure 5:**
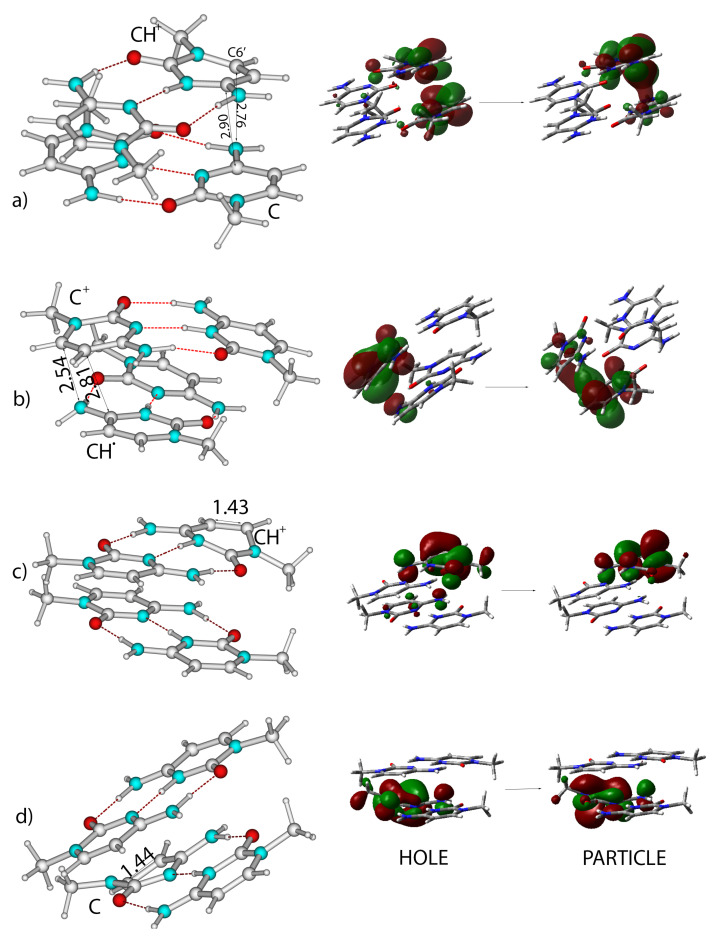
Schematic drawing of the most relevant minima and pseudo-minima obtained by optimizing the lowest energy excited states in C2-2. The description of the character of the excited states in terms of the involved NTO is shown on the left. (**a**) CH${}^{+}$-$\pi \pi_{CT}^{*}$-min, (**b**) C-CH${}^{+}$-CT-min, (**c**) CH${}^{+}\pi \pi^{*}$-min*, (**d**) C$\pi \pi^{*}$-min.

Geometry optimizations of S${}_{1}$ and S${}_{2}$ led to a minimum, mainly localized on the CH${}^{+}$ moiety, but with a non-negligible C→CH${}^{+}$ character. Indeed, while the particle was essentially localized on a CH${}^{+}$ base, the ’hole’ had some contribution from the stacked C base (see Figure 5a). We label the resulting minimum as CH${}^{+}$-$\pi \pi_{CT}^{*}$-min to highlight its partial CT character. However, this minimum was quite bright (oscillator strength ∼0.1), with emission energy $\lambda_{em}^{c}$∼410 nm. In this minimum, the two bases involved in the excited states became closer, with the -NH${}_{2}$ group of the C bases approaching the C5′ and C6′ atom of the CH${}^{+}$ base (see Figure 5a).

The coupling between CH${}^{+}$-$\pi \pi^{*}$ and the C→CH${}^{+}$ was large, and we did not succeed in locating a real minimum where the excitation was localized on a single CH${}^{+}$ moiety. However, by mapping the excited state surfaces, we found a flat region in the Potential Energy Surface (PES), where the energy gradient was small (∼0.001 a.u.) and the excitation was almost totally localized on CH${}^{+}$. In this pseudo-minimum (which we labeled as CH${}^{+}\pi \pi^{*}$-min*), which was very similar to that described in our parallel study of hemiprotonated (CH·C)${}^{+}$ pairs [69], the ring of the ’excited’ CH${}^{+}$ was no longer planar (see Figure 5c) and the C5′ = C6′ bond length increased. From CH${}^{+}\pi \pi^{*}$-min*, the system could decay, either to CH${}^{+}$-$\pi \pi_{CT}^{*}$-min (which was more stable by 0.20 eV) or to a crossing region with S${}_{0}$. This crossing region, which allowed a fast GS recovery, was separated by a very small energy barrier (<0.1 eV) from CH${}^{+}\pi \pi^{*}$-min*. This could be reached by the out-of-plane motion of the C5′-H5’ moiety, confirming the indications obtained in our study of hemiprotonated (CH·C)${}^{+}$ pairs [67].

Geometry optimization of S${}_{3}$ led first to the localization of the excitation on one of the C moieties, followed by decay to a close-lying C→CH${}^{+}$ CT state (C-CH${}^{+}$-CT, which is described just below) and to its minimum (C-CH${}^{+}$-CT-min). Nonetheless, we succeeded in optimizing a minimum where the excitation was localized on a C base (C$\pi \pi^{*}$-min, see Figure 5d). The ’excited’ C was indeed characterized by a strongly bent structure, with a significant lengthening of the C5=C6 bond involved in the electronic transition. As found for hemiprotonated (CH·C)${}^{+}$ pairs [67], a small out-of-plane motion of the H5 in the excited C, with an associated barrier ≤ 0.1 eV, leads to a crossing region with S${}_{0}$.

Geometry optimization of C-CH${}^{+}$-CT (S${}_{5}$) led to the C-CH${}^{+}$-CT-min minimum, already introduced above, where, due to the CT, the CH moiety was no longer positively charged but rather could be described as a neutral radical C-H${}^{\cdot }$, whereas the C bore a substantial positive charge (C${}^{+}$). C-CH${}^{+}$-CT-min is very weakly emissive (oscillator strength 0.02), with an emission energy $\lambda_{em}^{c}$ = 480 nm, and is slightly more stable (by ∼0.1 eV) than CH${}^{+}$-$\pi \pi_{CT}^{*}$-min. The two moieties involved in the CT state became closer than in the GS minimum. In particular, the distance between the N4’ amino group of one CH${}^{\cdot }$ base and the C6 atom of the stacked C${}^{-}$ base was only 2.5 Å, and the C4’ atom also became closer to the C5 one of the C${}^{-}$ base (see Figure 5b). At the same time, the C5-C6 bond length increased up to 1.42 Å and the C4’-N4’ to 1.38 Å. These are indications of a possible dimerization reaction between the two bases. Indeed, as shown in Figure 5b, the inspection of the NTO associated with this transition indicated that there was a bonding contribution between N4’ and C6 atoms, suggesting the possible formation of a new bond between these atoms. We focus on this reactive channel in Section 2.5.

The indications provided by our study of C2-2 were fully confirmed by the calculations performed on C2-4 and dC2-4 (see Supplemental Information (SI)), i.e., for longer sequences, including the backbone.

By optimizing S${}_{4}$, i.e., the most intense excited state in the GS minimum, we reach CH${}^{+}$-$\pi \pi_{CT}^{*}$-min. As detailed in the Supplemental Information (SI) (see Figures S11 and S12), the main features of CH${}^{+}$-$\pi \pi_{CT}^{*}$-min and C-CH${}^{+}$-CT-min minima are similar to that described above for C2-2.

### 2.4. Photophysical Decay Paths

**Figure 6:**
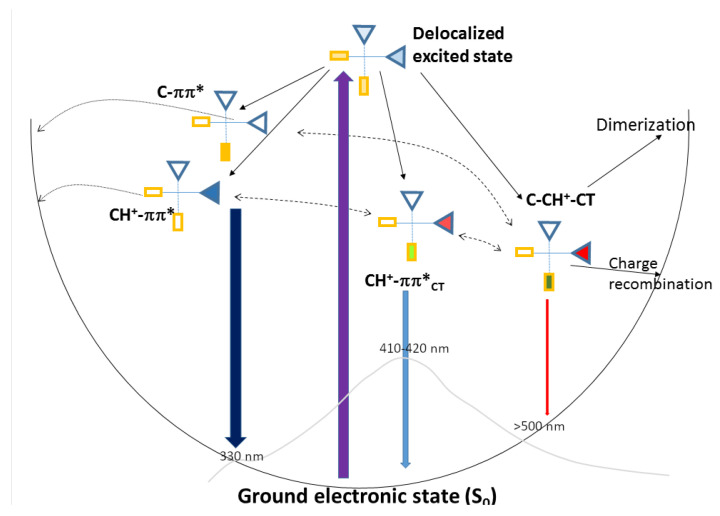
Schematic drawing of the possible decay paths occurring in the core of I-motifs. CH${}^{+}$ bases are represented as triangles, C bases as rectangles. The intensity of the color inside the bases is proportional to its participation in the excited state. The fluorescence spectrum of (dC)${}_{20}$ at acid pH [58] is sketched in gray.

In this section, based on the characterization of the excited state minima, we describe the main photophysical paths operating in the core of I-motifs and interpret the available steady-state and TR experiments. Concerning the emission, as sketched in Figure 6, I-motif-forming sequences exhibit a broad fluorescence spectrum peaking at 410∼420 nm, with a long red-wing tail until 700 nm, and a shoulder on the blue [58]. The fluorescence decay is strongly multi-exponential, with components from the sub-ps scale up to the ns [56,58]. The fluorescence quantum yield is one order of magnitude larger than that of protonated cytosine, i.e., a relatively small increase, considering the dramatic increase in the excited state lifetime [56]. Based on our calculations, we could assign the maximum of the fluorescence band to the emission from CH${}^{+}$-$\pi \pi_{CT}^{*}$-min. Not only was the latter the lowest-energy bright excited state, but the predicted emission energy was fully consistent with the experimental one. On the other hand, the weakly emissive C-CH${}^{+}$-CT-min likely contributes to the red-wing of the fluorescence spectrum. It is more stable than CH${}^{+}$-$\pi \pi_{CT}^{*}$-min and its stability can increase with time, due to the equilibration of environmental degrees of freedom, as suggested by the results obtained on other DNA sequences [45,70]. Indeed, the ‘readjustments’ of the solvent cage and, especially, of the phosphodeoxyribose backbone in response to the change in the electron density associated with the CT transition require a finite time [45,71].

Concerning ’monomer’-like emission from C-$\pi \pi^{*}$ and CH${}^{+}$-$\pi \pi^{*}$, the contribution of the strongly distorted minima to the fluorescence spectra is expected to be limited for two reasons: (i) these minima are less stable than CH${}^{+}$-$\pi \pi_{CT}^{*}$-min and C-CH${}^{+}$-CT-min; (ii) the energy barrier separating them from the crossing region with the GS is very small. On the other hand, it is possible that, in poorly stacked regions, the I-motif C and CH${}^{+}$ behave as ‘free’ bases and, therefore, emit at around 330 nm [68], thus contributing to the blue part of the fluorescence spectrum.

**Figure 7:**
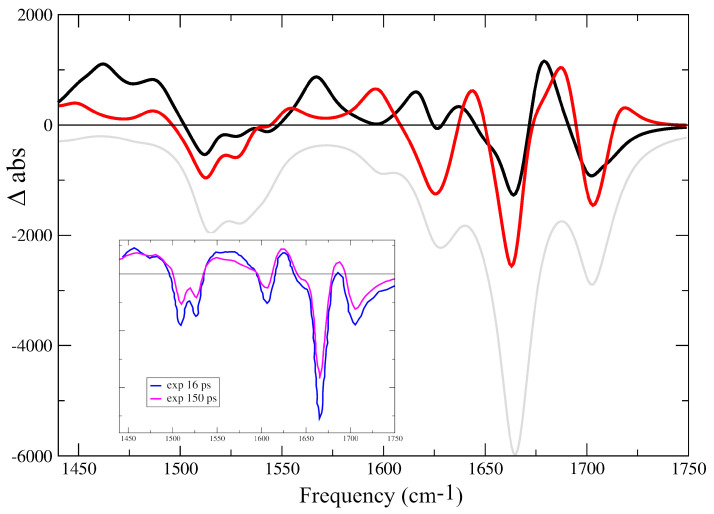
Computed difference IR spectra with respect to the ground electronic state for C2-4 associated with CH${}^{+}$-$\pi \pi_{CT}^{*}$-min (black line) and C-CH${}^{+}$-CT-min (red line). In the inset, we show the experimental DIR spectra measured for dC${}_{30}$ at pH 5.5, after 16 ps (blue line) and 150 ps (magenta line). IR frequencies scaled by 0.955. MC/TD-M052X/6-31G(d) calculations.

The TR-IR spectra of different sequences in I-motifs [55,72] exhibit a typical pattern. In addition to the bleaching bands due to the GS depletion, there are the characteristic decay features associated with the cooling of the vibrationally ‘hot’ ground states, related to the ultrafast GS recovery (within 4–5 ps) of the poorly stacked bases. As shown in the inset in Figure 7, there are positive bands, associated with the long-living excited states, lasting for hundreds of ps: one at 1450∼1470 cm${}^{-1}$, an intense one in the region 1540∼1580 cm${}^{-1}$ (with a maximum at 1574 cm${}^{-1}$), one at ∼1630 cm${}^{-1}$ and another one just below 1700 cm${}^{-1}$. Interestingly, the intensities of these two latter peaks, especially that on the blue-wing, increase with time (compare the blue and magenta lines in the inset in Figure 7). At the same time, the shape of the band in the range 1540∼1580 changes and the relative intensity of the 1574 cm${}^{-1}$ peak decreases.

Considering the approximations in our computational approaches and that, as stated above, in C2-2, the peaks of the GS are slightly blue-shifted with respect to those predicted for larger models, both CH${}^{+}$-$\pi \pi_{CT}^{*}$-min and C-CH${}^{+}$-CT-min have Difference IR (DIR) spectra with strong similarities to the experimental ones.

As reported in Figure 7, both exhibit positive Excited State Absorption (ESA) bands below 1500 cm${}^{-1}$ and in the 1540∼1580 cm${}^{-1}$ region. Both have positive peaks slightly below 1700 cm${}^{-1}$ and below 1650 cm${}^{-1}$. Some differences between the two DIR spectra are present. CH${}^{+}$-$\pi \pi_{CT}^{*}$-min has larger ESA at ∼1575 cm${}^{-1}$ and two peaks close to 1625 cm${}^{-1}$, whereas the peak in C-CH${}^{+}$-CT-min below 1700 cm${}^{-1}$ is blue-shifted, and two peaks appear at ∼1600 and ∼1650 cm${}^{-1}$. The comparison between experimental and computed DIR spectra suggests that both C-CH${}^{+}$-CT and CH${}^{+}$-$\pi \pi_{CT}^{*}$ coexist for several dozen picoseconds, with the relative population of C-CH${}^{+}$-CT increasing with time (see the Discussion section).

The difference spectra of CH${}^{+}$-$\pi \pi_{CT}^{*}$-min and C-CH${}^{+}$-CT-min computed for C2-4 are consistent with those described in detail above (see Supplemental Information (SI)).

### 2.5. Photochemical Paths

**Figure 8:**
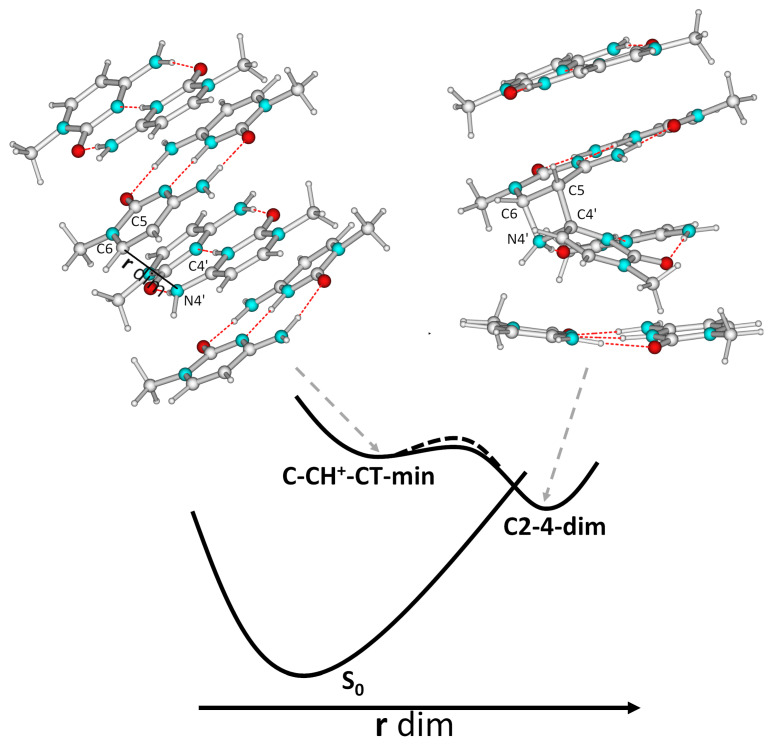
Schematic description of the minimum energy path associated with the formation of C2-4-dim on the PE of C-CH${}^{+}$-CT. The structures of C-CH${}^{+}$-CT-min and C2-4-dim are also shown. The dashed lines refer to the energy barrier predicted for the same path for dC2-4.

IR experiments indicated that UV irradiation of different sequences of folding in I-motifs can induce the formation of photodimers [57]. As anticipated above, the two bases involved in the CT transition are very close in C-CH${}^{+}$-CT-min. As a consequence, we investigated if a dimerization reaction could occur along this path by computing a minimum energy path for the formation of a bond between the amino nitrogen atom of the CH${}^{+}$ moiety (N4’) and the C6 atom of the stacked C base (**r dim** in Figure 8).

Our calculations indicate that, after the very shallow C-CH${}^{+}$-CT-min, the system passes through a plateau, and, after overcoming a very small energy barrier (≤0.05 eV), it can reach a crossing region with S${}_{0}$ (S${}_{1}$/S${}_{0}$ energy gap ≤ 1 eV). In that region, the distance between N4’ and C6 is ∼1.60 Å, indicating the incipient formation of an N-C bond. At the same time, the C5-C6 bond distance increases up to 1.48 Å and the C4’-C5 reaches 2.6 Å, suggesting the formation of a four-member ring. TD-DFT is not suitable to describe the crossing region with S${}_{0}$ in detail, so we could not fully characterize it. In any case, we mapped the PES of the dimerization path by DFT GS geometry optimization, which indeed indicated the formation of a stable CH${}^{+}$/C dimer (C2-4-dim, see Figure 8), which was more stable by ∼2 eV than the crossing region.

**Figure 9:**
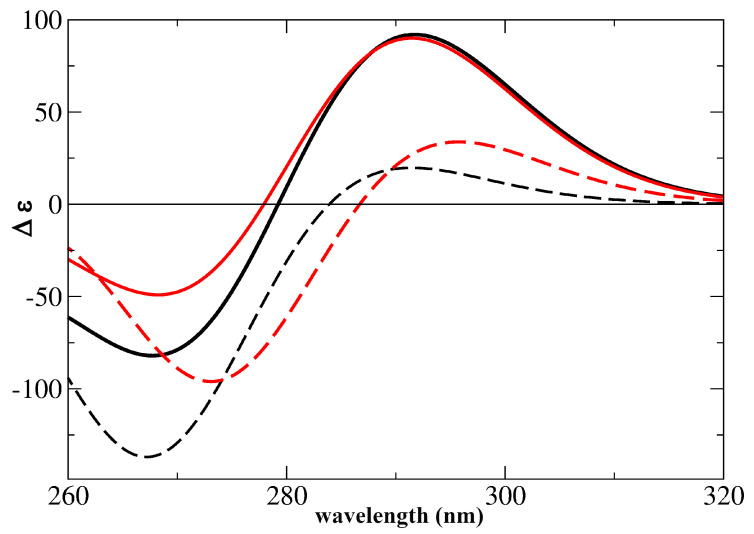
ECD spectra computed for C2-4 (black continuous lines) and dC2-4 (red continuous lines) compared with those computed for C2-4-dim (dashed black lines) and dC2-4-dim (dashed red lines). TD-PCM/M052X/6-31G(d) transition shifted by −0.6 eV and broadened with a Gaussian with HWHM = 0.2 eV. Δϵ expressed in 10${}^{-40}$ esu${}^{2}$ cm${}^{2}$.

We characterized the ECD and the IR spectral signatures of C2-4-dim, comparing them with those of C2-4. As shown in Figure 9, the shape of the computed ECD spectrum was very similar to that of C2-4; no significant shifts in the position of the maxima were found, but the intensity of the positive peak significantly decreased. This outcome is consistent with experimental results, which do not show any shift in the ECD band, but only a decrease in their intensity after UV irradiation of some I-motif-forming DNA sequences [57]. As discussed in the Supplemental Information (SI), Section S2.2, the IR spectral features of C2-4-dim were also consistent with the experimental indications [57].

We also analyzed the possible dimerization for dC2-4, in order to verify how this process is affected by the backbone. We obtained a similar picture to that just described for C2-4, but a large energy barrier, i.e., ∼0.2 eV, was associated with the path leading to the formation of the dimer (dC2-4-dim). As shown in Figure 9, the ECD features of dC2-4-dim are similar to those of C2-4-dim.

## 3. Discussion

A complete disentanglement of the photoactivated dynamics in an NA sequence arranged in an I-motif poses tremendous challenges to a quantum mechanical treatment. The core, formed by a (CH·C)${}^{+}$ pair, includes at least 8 bases (i.e., ≥100 atoms), without considering the phospho-(deoxy)ribose backbone. Moreover, ∼50 excited states fall in the lowest-energy absorption band, that is responsible for the typical ECD signature of I-motifs, making a complete characterization of the possible stationary points and crossings impossible. Thus, we should consider that the spectral features of I-motifs are also affected by the thermal fluctuations in the chain and by the bases of the loops. These features pose some limitations on the choices of the computational approaches to be used, making it, for example, much more convenient to describe solvent effects by a continuum model (i.e., it is almost necessary to resort to DFT and TD-DFT) and to discard anharmonic contributions to IR spectra. In order to overcome, at least partially, these limitations, we herein focused on the (CH·C)${}^{+}$ core of the I-motif (which is expected to be more rigid than loops), analyzing several sequences which differed in size, protonation patterns and complexity (i.e., including or excluding the backbone), by using different computational approaches. In this way, we aimed to obtain a picture as complete and solid as possible, at least within the scope of this study, i.e., to provide a global, yet sometimes qualitative, picture of the photoactivated dynamics in I-motifs, interpreting all the main experimental evidence available—not a trivial task. TR Experiments on I-motifs indeed showed complex excited state decay mechanisms, with signals that strongly depended on the wavelength considered and the signature of several different photophysical and photochemical processes. In the following paragraphs, we discuss the main outcomes of our analysis.

The typical IR and ECD signature of I-motifs. Our computational approach reproduces the typical changes in the shape of steady-state IR, ECD and UV spectra associated with the formation of I-motifs at acidic pH very well. Additionally, based on our analysis, two main factors determine the spectral properties of I-motifs: (i) the presence of protonated cytosine and hemiprotonated (CH·C)${}^{+}$; (ii) their arrangement in intertwined chains. It is not easy to disentangle the relative importance of these effects because they also depend on the structure adopted by the same sequence at neutral pH, where several conformations can be in equilibrium. Although assessing the conformational behavior of C-rich sequences at neutral pH falls outside the scope of this study, we have studied two limiting cases. In one, the C bases keep an I-motif-like structure and, in the other, they adopt a B-DNA-like single strand (see Supplemental Information (SI), Section S2.1 and Figure S1). As discussed in the Supplemental Information (SI) (Section S2.2), the IR signature depends, essentially, on the presence of hemiprotonated (CH·C)${}^{+}$ pairs. Even when adopting an I-motif-like structure, hydrogen-bonded Cs are characterized by an IR spectrum which is very different from that of I-motifs at acidic pH. On the other hand, it is important to remind the reader that an I-motif-like structure can stabilize the protonation of C, even at a pH higher than the pK${}_{a}$ of cytidine.

The ECD spectrum depends, instead, on the ‘I-motif like’ arrangement. Experiments indicate that its peculiar shape is lost when the formation of the I-motif is prevented by increasing the pH or, on the contrary, by decreasing to a value for which all the Cs are protonated [29,30,39,40,56]. Our calculations also indicate that at least 3/4 (CH·C)${}^{+}$ pairs are necessary to obtain a spectral shape which is consistent with the experimental ones. Finally, the possibility of different hemiprotonation patterns does not affect the ECD spectra, but constitutes an additional source of broadening. As discussed in detail in the SI, we tried to obtain some additional insights on these phenomena by simulating the ECD spectrum of two limit structures where all the C were not protonated, and we obtained some interesting indications. For example, as shown in Figure S7 in the Supplemental Information (SI), the ECD spectrum of C bases, arranged as in an I-motif, shows similar trends to that observed when increasing the pH in C-rich sequences, which should be characteristic of unstructured forms [40].

Photophysical decay paths and fluorescence. Our calculations indicate that the absorption of UV light leads to the population of delocalized excited states on more than one base (see Figure 6). According to our results, concerning their photophysics, I-motifs can be considered as a collection of CH${}^{+}$ and C bases. In other words, the presence of a ‘shared’ proton in [CH·C]${}^{+}$ does not induce particular photophysical features. In fact, the energy barrier associated with the hopping of the proton between between the two C bases is not very small, and the rate of this process (10${}^{8}$ s${}^{-1}$) [73] is too slow to affect ultrafast photophysics. We have, thus, identified two families of bright excited states that contribute to the lowest-energy absorption band. The red-wing is associated with the excitons resulting from the coupling between the lowest-energy bright excited states’ localized CH${}^{+}$, whereas those deriving from the bright excited states of C bases contribute to the blue-wing. On the other hand, the presence within the I-motifs of several closely stacked [CH·C]${}^{+}$ dimers leads to the appearance of C→CH${}^{+}$ CT states, which can mix with the bright transition. In the S${}_{0}$ minimum, the coupling is quite small and, as a consequence, we can identify states with a clear-cut C→CH${}^{+}$ CT character 0.3∼0.5 eV (C-CH${}^{+}$-CT) on the blue with respect to the bright states, whose CT character is quite small. However, when considering the effect of thermal fluctuations, we would find structures for which the couplings between C-$\pi \pi^{*}$, CH${}^{+}$-$\pi \pi^{*}$ and C→CH${}^{+}$ CT states are larger than what is predicted in the FC point. As a matter of fact, when optimizing the excited states, we find that the one of two most stable excited state minima has a strong mixed CH${}^{+}$-$\pi \pi^{*}$/C-CH${}^{+}$-CT character. As depicted in Figure 6, this minimum (quite bright) is associated with the maximum of the fluorescence spectrum of I-motifs at 410∼420 nm [58]. The most stable excited state minimum is associated with C-CH${}^{+}$-CT, with red-shifted emission but low intensity, mirroring its significant CT character, which should contribute to the red-wing of the experimental spectrum and be involved in the photochemical path discussed below. Actually, as sketched in Figure 6 by the dashed double arrow line, C-CH${}^{+}$-CT-min and CH${}^{+}$-$\pi \pi_{CT}^{*}$-min can be considered the minima associated with two limiting cases of an excited state deriving from the coupling of a bright state localized on CH${}^{+}$ and the CT transition. The most realistic picture of the situations experienced in a DNA/RNA sequence folded in an I-motif is that of an excited state that, depending on the environmental fluctuations (backbone, of the solvent, counter-ions) and on the conformational behavior of the loops, can have a different degree of CT character and, therefore, slightly different spectral properties. On the other extreme, for poorly stacked bases, decay to localized excited states, associated with emission in the blue, e.g., below 350 nm, is possible.

The existence of a dynamic equilibrium between different excited states, which has been suggested to be operative for other DNA sequences [45,51,70], can explain the puzzling dynamical behavior of I-motifs, as evidenced by TR experiments. Considering the presence of possible emission minima, each one with different features, we can, thus, explain why TR fluorescence is strongly wavelength-dependent, decaying more slowly when the emission wavelength increases. The emission at 330 nm decreases mainly on the sub-ps time-scale, whereas the emission at 500 nm, which we associate with CT exciplexes, decays more slowly [58], with the largest component being ∼60 ps. At the same time, when exciting the blue of the absorption band associated with ‘monomer-like’ channels, the decay is, on average, faster [56,58]. Finally, the weight of long-living species (on the ns time-scale) is larger in transient absorption spectra (which can also monitor non-emissive species, such as C-CH${}^{+}$-CT-min) than in TR fluorescence spectra [56].

Photochemical paths. Our calculations indicate that, along the PES of C-CH${}^{+}$-CT, a quite accessible path towards the formation of a dimer exists. It is similar to that involved in the formation of the 6-4 pyrimidine–pyrimidone (6-4) photoproduct (6-4PP) in bi-pyrimidine steps [43,45,69,74], which has been suggested to occur on the PES of the CT state from the pyrimidine in the 5’-end position towards that on the 3’-end [45,69]. Interestingly, when this reaction involves a TpT or a dCpT step, it proceeds through an oxetane intermediate [45,69,74]. In the case of TpdC, QM calculations indicate that, though it could, in principle, involve an azetidine intermediate, it proceeds in a single step [69]. In our case, likely due to the participation of a protonated C-H${}^{+}$, a stable four-member intermediate is predicted (see Figure 8), whose spectral IR and ECD features seem compatible with experimental indications [39].

As discussed in the Supplemental Information, we further investigated this reaction, identifying two additional photoproducts that can be formed, starting from C2-4-dim. These species are characterized by ECD and IR spectra which are less consistent with the experimental ones with respect to C2-2-dim (see Figures S13–S15 in the Supplemental Information (SI)), but their formation cannot be discarded.

It is clear that this analysis can be considered only a first step toward a full characterization of the photodimerization path, which could be, in principle, significantly affected by the interaction with solvent molecules, by possible small rearrangements of the backbone. It is also possible that our estimates provide a lower bound for the energy barrier towards the dimerization and that the the products we have discussed are formed with a lower quantum yield than the other ones potentially formed in the oligonucleotides studied in the experiments. It is indeed important to remind the reader that other photodimers, also involving the bases in the loops, can be produced in the sequences which form I-motifs [57]. Moreover, we did not consider the possible effect of the partial unfolding of the I-motifs, which could also affect the shape of the ECD and IR spectra recorded after photodimerization [57].

## 4. Materials and Methods

A detailed description of our computational models is reported in Section S1 of the Supplemental Information (SI), where we also discuss more extensively their possible limitations, which are also highlighted in the first paragraph of the Discussion (Section 3).

Computational Models. Our reference computational model, shown in Figure 1c, was built by using the NMR solution structure of a modified human telomere fragment (1ELN.pdb) [15] as starting geometry, limiting the central cluster to four (CH·C)${}^{+}$ hydrogen-bonded dimers formed by four intercalated (dC)${}_{2}$ dinucleotides (hereafter dC2-4) and by adopting a 3’E topology [4]. Test calculations (Section S2.3 of the Supplemental Information SI) were performed for tautomers by differing the location of the ’extra’ proton. We also studied computational models including only the C bases (hereafter, C2, C2-2, C2-4 and C2-4, see Figure 1d) by using a methyl group to mimic the sugar [68]. These models, in addition to reducing the computational cost, allowed us to study the dependence of the properties of the I-motif on the number of (CH·C)${}^{+}$ hydrogen-bonded dimers. In the following sections, when necessary, we label the atoms of the CH${}^{+}$ moiety with a prime.

Electronic method. Due to the size of the systems under study (≥150 atoms) and the necessity to consider dozens of excited states, we used Density Functional Theory (DFT) and its time-dependent extension (TD-DFT). Concerning the critical choice of the density functional, as also explained in the SI, based on our experience in the study of DNA photophysics [45,51], we selected functional M052X [75], which provides a fairly accurate description of stacking interactions and the relative stability of Charge Transfer (CT) transitions [75], as the reference. We profitably used M052X to study the photoactivated behavior and the spectra from different NA sequences arranged in single, double and quadruple strands [45,51,52,76,77,78], always obtaining indications fully consistent with those obtained on smaller models with more accurate ab initio methods. In a very recent study, we indeed showed that this accurately describes the most relevant spectral features of a protonated cytidine and hemiprotonated (CH·C)${}^{+}$ pair, i.e., the building blocks of the I-motif, in good agreement with results obtained at the CASPT2 level or by using other long-range corrected density functionals [67]. The reliability of M052X predictions was checked (Section S2.3 in the Supplemental Information (SI)) via some tests with two other commonly used long-range corrected functionals, i.e., CAM-B3LYP [79] and ωB97XD [80]. Most of our analysis has been performed with the cost-effective 6-31G(d) basis set, checking the reliability of its predictions by performing test calculations with larger basis sets, such as the 6-311G(d,p) one (see Section S2.3 of the Supplemental Information (SI)). Bulk solvent effects have been included using the Polarizable Continuum Model, PCM [71].

Simulation of the spectra. The spectral shapes were simulated by simply convoluting the ‘stick’ contribution of each excited state by a Gaussian, with half-width–half-maximum (HWHM) = 0.2 eV. In order to enable a more direct comparison with experiments, we report spectra red-shifted by 0.65 eV, corresponding to the difference between the maxima of the experimental absorption band of C and CH${}^{+}$ and vertical transition energies computed at our level of theory [67]. This discrepancy was partially (0.1∼0.2 eV) due to lack of thermal and vibrational effects in our treatment [81,82] and in large part due to the limitations in our computational approach (e.g., density functional, basis set, solvation model). However, we have shown that the differences between the computed and experimental spectra in oligonucleotides are due to the errors in the description of the monomer and not of the inter-bases electronic interactions [77,78].

IR spectra were computed after substituting all of the hydrogen atoms bonded to nitrogen and oxygen atoms with deuterium ions in the Harmonic approximation, scaling each frequency by 0.955 in order to account of the lack of anharmonic effects [83,84]) and then broadening each stick transition with a Lorentzian with HWHM = 10 cm${}^{-1}$.

All calculations were performed with the Gaussian16 package [85].

## 5. Conclusions

We here tried to provide as complete of a picture as possible of the processes triggered by UV irradiation of the ‘core’ of I-motifs, from absorption to emission, while also considering the possible photochemical reactions. Based on our calculations, we can assign and interpret the main spectroscopic evidence, both steady-state and time-resolved, available in the literature. Though we have studied a minimal model of I-motifs, formed by four (CH·C)${}^{+}$ pairs (dC2-4), several indications suggest that our results can provide a reliable basis to interpret the behavior of longer sequences and to define the underlying chemical–physical effects. For example, a very recent study shows that, in long I-motif-forming sequences, structures with a small number of (CH·C)${}^{+}$ pairs are formed very rapidly and can act as kinetic traps for the formation of longer more ordered sequences, which occurs on a time-scale much slower than that typical of biological processes [30]. Interestingly, the smallest sequence that can be formed could be the one we have studied here, i.e., dC2-4, suggesting that the results we report here could be a useful basis for interpreting the behavior in longer, more biologically relevant sequences. Moreover, our simulated spectra (IR, absorption, fluorescence, ECD) are in good agreement with the experimental ones of larger sequences, suggesting that the (CH·C)${}^{+}$ core plays a dominant role in determining the photoactivated dynamics of I-motifs and that our model is sufficiently large to capture their ‘physics’.

Overall, the general picture of the the photophysical behavior in I-motifs is similar to that obtained in other DNA structures [45,51]. Even if, in I-motifs, the bases in adjacent steps are more poorly stacked than in duplex and quadruplex DNA strands, the most-populated decay pathways involve two ‘stacked’ C and CH${}^{+}$ bases, participating in excited states with a certain degrees of CT character—smaller for CH${}^{+}$-$\pi \pi_{CT}^{*}$ and larger for C-CH${}^{+}$-CT. This outcome enables an additional step towards the attainment of a unifying picture of the photoactivated dynamics in nucleic acids [45,51]. At the same time, since the main deactivation routes involve only two (CH·C)${}^{+}$ pairs, we are confident that our conclusions are also valid for I-motif sequences longer than those investigated here. In addition to the possible contribution of ‘monomer-like’ decay paths, we thus attribute the complex excited state decay, which is strongly dependent on the wavenlength, to the interplay between CH${}^{+}$-$\pi \pi_{CT}^{*}$ and C-CH${}^{+}$-CT, as revealed by TR experiments [55,56].

We have shown that a relatively accessible dimerization path exists on the PES of C-CH${}^{+}$-CT, leading to the formation of a thermodynamically stable photodimer, C2-4-dim, involving a stacked C and CH${}^{+}$ bases, in a reaction similar to that producing 6-4 PP dimers in bi-pyrimidine steps (see Figure 8). We have also described some of the reactive paths originating from C2-4-dim, which can also produces a 64-PP dimer involving two C bases (see Figure S14 in the Supplemental Information (SI)). These new insights on the photophysics/photochemistry of I-motifs can be relevant, not only for the study of the oxidative damage in DNA, but also for any applications of I-motifs exploiting light irradiation. To make an example, there are experimental indications of the interaction between the I-motif cores and the photoexcited labels used in biosensing applications [26]. Moreover, the degree of CT character in the different excited state minima can be sensitive to the conformational behavior of the backbone, modulated by the loops, by the presence of ligands, etc. On this basis, we can envisage the possibility of modulating the spectral properties, for example, the fluorescence or the excited state lifetime of an I-motif, with small modifications to the sequence and/or of the external conditions. Finally, the formation of dimers could affect the stability and the performance of I-motif sequences in nanotechnology applications.

On the other hand, it is clear that there are a number of questions arising from the findings reported here. For example, concerning the photochemistry of I-motifs, it will be important to assess the biological relevance of the photodimer(s) that we have shown can be formed, to determine their ‘final fate’, verifying if their formation is competitive with that involving the base of the loops and if the I-motif structure hampers the repair machinery.

More generally, any advance in our understanding of the excited state behavior of the I-motif ‘core’ is only a first step towards a full harnessing of the properties of the real DNA/RNA sequences which can adopt these particular structures. A key issue to be tackled concerns the role played by the bases in the loops. Loops have been shown to noticeably affect that spectral properties of I-motifs [34]. Loop bases can directly contribute to the spectroscopic signal [34], but their effect could also be indirect, i.e., related to the conformational restraint induced in the I-motif core. For example, we show here that C2-4 and dC2-4 ECD spectra, though similar, are different, and this is partially due to the small differences in the stacking geometry induced by the backbone. Our calculations suggest that another, more subtle, effect is possible, related to the possible influence of the loop on the hemiprotonation pattern in the I-motifs. The location of the ‘extra’ proton is indeed governed by the maximization of their distance [73], which is expected to be affected by the asymmetries related to the structural behavior in the loops.

The bases of the loops surely modulate the entire conformational behavior of the I-motif, including their unfolding and refolding processes, another hot topic in this field [30,33]. There are convergent indications that the folding and unfolding of I-motifs involve different intermediates [26,30,86] It is, thus, likely that, for the majority of C-rich sequences, an equilibrium between I-motifs, also with a varying number of (CH·C)${}^{+}$ pairs, hairpins and duplex structures, is operative [38,87]. Additional information on the structural behavior in C-rich regions is, thus, critical. In this respect, despite being focused only on the structured ‘more rigid’ core of I-motifs, this study provides promising perspectives for the study of the conformational dynamics in I-motifs. For example, we have shown that minimal structures, with only two (CH·C)${}^{+}$ pairs, can induce an increase in the ellipticity at ∼290 nm, even if their spectrum is quite different with respect to that of I-motifs; we have also shown that ‘I-motif-like’ arrangements of non-protonated cytosines can produce an ECD spectrum similar to that of unstructured sequences. Quantum mechanical calculations are powerful investigative tools to translate spectral signals into a well-defined structural picture at the atomic level. On the other hand, when dealing with flexible structures such as those forming I-motifs, proper inclusion of thermal fluctuations is important, requiring the integration with MD simulations [33,88] and, in order to simulate the spectra, with cost-effective approaches such as those based on excitonic hamiltonians [76,89]. We have recently profitably adopted these procedures in the study of DNA duplexes and quadruplexes [76,77,78] and we plan to extend them to the study of I-motifs. Hopefully, in the near future, combining the information gained in this latter way with that provided by time-resolved spectroscopy [55,58,72], multivariate analysis [34,39,87] and MD simulations [33], it will be possible to achieve a more complete understanding and control of the processes involving I-motifs.

## Data Availability

All data are provided in the results section and the Supplemental Material accompanying this paper. The outputs of the calculations made during the current study are available from the corresponding author on reasonable request.

## Supplementary Materials

The supporting information can be downloaded at: https://www.mdpi.com/article/10.3390/ijms241612614/s1. References [90,91,92,93,94] are cited in Supplementary Materials.
