# Supplementary material for: Shedding Light on the Photophysics and Photochemistry of I-Motifs Using Quantum Mechanical Calculations
Source: Int J Mol Sci. 2023 Aug 9;24(16):12614. doi: 10.3390/ijms241612614 (PMC10454157; doi:10.3390/ijms241612614)
Supplement: Supplementary file 1 [file ijms-24-12614-s001.zip › ijms-2541246-supplementary.pdf]

Shedding light on the photophysics and the photochemistry of DNA I-motifs by quantum mechanical calculations.
Supplemental Information

Roberto Improta

Consiglio Nazionale delle Ricerche, Istituto di Biostrutture e Bioimmagini (IBB-CNR), Via De Amicis 95,I-80145 Napoli, Italy; roberto.improta@cnr.it

S1 Additional Computational Details

The computational study of the photoactivated behavior of an I-motif forming DNA sequence is extremely challenging. The description of excited electronic states requires the use of computationally expensive quantum mechanical calculations. Moreover, the size of the systems under study ($\geq$ 150 atoms) and the necessity to consider dozens of excited states pose strict requirements for the choice of the reference computational method, making the methods rooted in Density Functional Theory (DFT) and its Time-Dependent extension (TD-DFT) the only possible option. TD-DFT has well-known weaknesses, for example in the treatment of the electronic transitions with CT character[1, 2], an important deficiency when treating closely stacked multichromophore systems as I-motifs. However, it has been shown that a suitable choice of the functional allows a reliable description of CT transitions.[2, 3] As a consequence the choice of the functional strongly affects the accuracy of the results. Then, it is necessary to consider solvation effects, since all the reference experiments are performed

in water. When simulating the spectra, vibrational effects should be in principle explicitly included, since they determine the spectral lineshape.[4] This is an impossible task when dealing with dozens of excited states, with strong non-adiabatic couplings potentially existing among them.[5]

In the following paragraphs, we shall thus describe in detail the different features of our computational approach, explaining the rationale of our choices.

Computational Models. Our reference computational model, shown in Figure 1c of the main text, has been built using as starting geometry the NMR structure in solution of a modified human telomere fragment (1ELN.pdb),[6] limiting to the central cluster of four $(CH{\cdot}C)^+$ hydrogen-bonded dimers, formed by four intercalated $(dC)_2$ dinucleotides (hereafter dC2-4), adopting an 3'E topology.[7] Since the 'extra' proton can reside on each of the C bases in the $(CH{\cdot}C)^+$ pair, multiple tautomers are possible for our computational model, differing for the location of the 'extra' proton. Due to the structural asymmetry related to the presence of the backbone, the different tautomers can have slightly different energies. As a consequence, as reported below in Section S2.3), we made some test calculations for four tautomers, differing in their 'hemi-protonation' pattern. We have also studied computational models including only the C bases (hereafter C2, C2-2, C2-4, and C2-4, see Figure 1d in the main text), which, besides reducing the computational cost, allow us to study the dependence of the properties of the I-motif on the number of $(CH{\cdot}C)^+$ hydrogen-bonded dimers. In these species, a methyl group is used to mimic the sugar since we have shown that this is a cost-effective recipe to study the excited state behavior of cytidine.[8]

Electronic method. Among the many density functionals available, in this study we choose as the reference functional M052X,[3] which provides a fairly accurate description of stacking interactions and of the relative stability of CT transitions.[3] Both features are critical when studying a closely stacked multichromophore system as DNA. In particular, accurate treatment of weak non-bonding interactions is very important for an accurate determination of the minima, both in the ground and in the excited electronic states. This choice is supported by our experience in the study of DNA,[9, 10], where we have profitably used M052X to study the photoactivated behavior of different NA sequences, arranged in single, double, and quadruple strands.[9–11] In several studies we have also benchmarked M052X against

the results obtained by using other ab-initio methods (CASPT2, EOM-CCSD, and so on) or other long-range corrected density functionals, always obtaining positive indications.[9, 10] For what concerns the present study, we have recently shown that it provides an accurate description of the most relevant excited state features of protonated cytidine and hemiprotonated $(CH{\cdot}C)^+$ pair, i.e. the building blocks of I-motif, in good agreement with that obtained at the CASPT2 level or by using other long-range corrected density functionals.[12]

On the other hand, as discussed below, M052X overestimates (by ~0.6 eV) the excitation energy of the bright states of nucleobases. However, it can very accurately reproduce the effect of the stacking on the spectra (IR, absorption, ECD) of DNA.[10, 11, 13–15] This feature is the most critical one for the aim of this study. In any case, as reported in section S2.3 below, the reliability of M052X predictions has been checked with some tests with two other commonly used long-range corrected functionals, i.e. CAM-B3LYP[16] and ωB97XD[17].

Most of our analysis has been performed by using the cost-effective 6-31G(d) basis set. However, we assessed the accuracy of its predictions by performing test calculations by using larger basis sets, such as the 6-311G(d,p) one (see Section 2.3 of the SI).

Solvent effects. We resorted to the Polarizable Continuum Model, PCM,[18] to include bulk solvent effects. Based on our previous experience on duplexes and quadruplexes[10], we have not explicitly considered solvent molecules in our models, since interbase hydrogen bonds strongly reduce the number of possible solute-solvent hydrogen bonds.

Simulation of the spectra. In order to compute the absorption and the ECD spectra of our I-motif models in the UVB-UVC region, it is necessary to compute ≥ 40 excited states of species containing hundreds of atoms. Proper inclusion of vibrational and thermal effects in the spectral lineshapes[4] is thus unfeasible. The spectra are thus simulated by simply convoluting the 'stick' contribution of each excited state by a Gaussian, with half-width-half-maximum (HWHM)=0.2 eV. M052X overestimates the transition energies of CH^+ and C by 0.6~0.7 eV, with respect to the maxima of the experimental absorption band.[12] This discrepancy is partially (0.1~0.2 eV) due to thermal and vibrational effects (not included in our treatment), which have been shown to systematically red-shift band maxima with respect to the vertical transition energies.[4, 19]. The largest part of this error is, however, due to the limitations of our computational approach (e.g. density functional, basis set, solvation

model). In order to enable a more direct comparison with experiments, we shall thus report spectra red-shifted by 0.65 eV, corresponding to difference between the maxima of the experimental absorption band of the monomers and vertical transition energies computed at our level of theory.[12] As a matter of fact, the test calculations performed in our previous studies indicate the differences between the computed and experimental spectra in oligonucleotides are due to the errors in the description of the monomer and not of the inter-bases electronic interactions.[14, 15]

IR spectra have been computed, after substituting with deuterium atoms all of the hydrogen atoms bonded to nitrogen and oxygen atoms, in the Harmonic approximation. The lack of anharmonic effects leads to an average blue-shift of the spectra. We thus resorted to an empirical procedure, commonly used in the literature, (see, for example,[20, 21]), scaling each frequency by 0.955, and then broadening each stick transition with a Lorentzian with HWHM=10 cm^{-1}.

S2 Additional Computational Analysis

S2.1 Non-protonated sequences

Starting from the I-motif minimum of C2-4, we have determined the ground state minimum of a structure where all C bases are not-protonated at N3. As shown in Figure S1, the structure keeps an I-motif-like arrangement, but there is a shift in the hydrogen bonding pattern of the CC pairs, with the amino group of each base hydrogen bonded to the N3 atom of its partner. Based on the similarity of the C2-4 structure, we shall label this structure as C2-4-neu, to highlight that it is aimed to mimic a neutral pH. In order to get insights into the behavior of C-rich sequences that are not able to properly fold in an I-motif like structure, because they are too short, for example, we have also optimized the geometry of a dC_6 oligonucleotide in a B-DNA arrangement (dC_6-neu). In this case, we started from the structure, in B-DNA conformation, reported in ref.[22], optimized at the DFT level.

This analysis is very far from being exhaustive. The conformation adopted by C-rich sequences will depend on the exact sequence, the number, and the position of the C tracts, and, at room temperature, the co-existence of different conformations is possible. Our aim

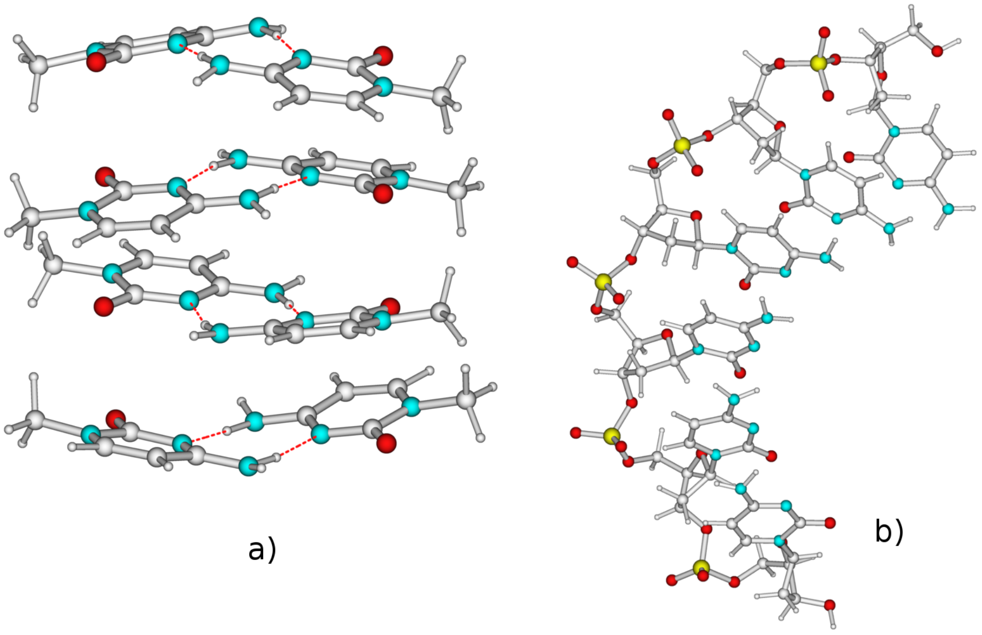

Figure S1: Schematic drawing of a) structure formed by 8 C bases arranged as within an I-Motif (C2-4-neu) b) $(dC)_6$ ($(dC)_6$-neu) Color code: C (grey), H (white), O (red), N (light blue), P (yellow)

is simply to make a first step toward the characterization of the possible spectral signature of the different arrangements (see the next subsections).

S2.2 IR spectra

In Figure S2 we compare the IR spectra computed for C2-4 and C2-4-neu, reporting also the experimental IR spectra computed for $(dC)_{30}$ at acidic and weakly basic pH.[23] Please con-sider that at weakly basic pH $(dC)_{30}$ is expected to adopt multiple conformations, including tracts in a single strand, for which the explicit inclusion of the hydrogen bonds with the solvent is more important. Considering also the other approximations in our computational model (e.g. lacking the phosphodeoxyribose backbone and not including thermal fluctua-tions), the computed scaled harmonic spectra are in good agreement with the experimental ones, for what concerns both the position and the relative intensity of the different bands. The most significant discrepancy with respect to the experiment concerns the overestimation of the intensity of the band at 1500~1550 cm^{-1}. However, the most important result of this analysis is that, as anticipated in the main text, our calculations well capture the two most

important effects of the hemiprotonation on the IR spectrum: the appearance of the band at $\sim$1700 cm^{-1} and the strong decrease of the intensity in the band at 1500$\sim$1550 cm^{-1}.

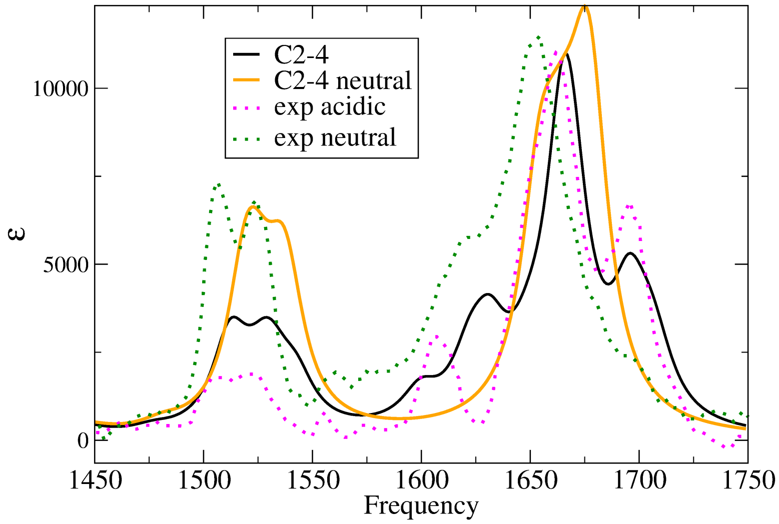

Figure S2: Computed IR spectra for C2-4 (black line) I-motif, for a species mimicking C2-4 at neutral pH (orange line). PCM/M02X/6-31G(d) calculations. Spectra simulated by multiplying each frequency by 0.955 and convoluting with a Lorentzian with hwhm=10 cm^{-1}. The experimental spectra measured for dC_{30} at pH=5.5 (dotted magenta line) and at pH 8.5 (green dotted line) are also shown.[23] Frequency in cm^{-1}, ϵ in L M^{-1} cm^{-1}.

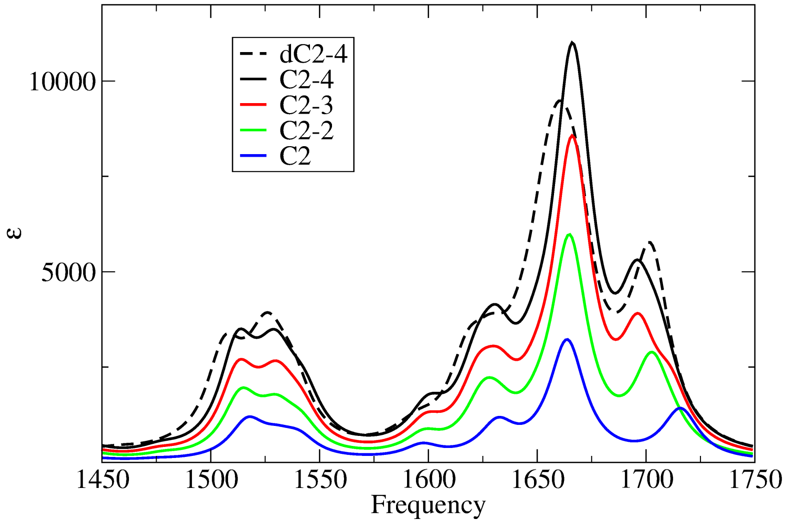

Figure S3: Computed IR spectra for C2-4 (black line) dC2-4 (black dashed lines), C2-3 (red line), C2-2 (green line), C2 (blue line) PCM/M052X/6-31G(d) calculations. PCM/M02X/6-31G(d) calculations. Spectra simulated by multiplying each frequency by 0.955 and convoluting with a Lorentzian with hwhm=10 cm^{-1}. Frequency in cm^{-1}, ϵ in L M^{-1} cm^{-1}.

In Figure S3 we show how the IR spectrum changes when the number of hemi-protonated

pairs considered increases. The most important change is a weak red-shift of the high-energy peak with the number of pair. However, the spectral shape does not dramatically change, suggesting that the presence of hemi-protonated pairs is more important than their folding within an I-motif.

S2.3 Absorption and ECD spectra

In this subsection, we report additional computational results concerning the absorption and ECD spectra. The spectra reported in Figure S4 show that the inclusion of the backbone in the computational model affects has a quite small effect on the computed spectral shape, only a weak red-shift of the maximum.

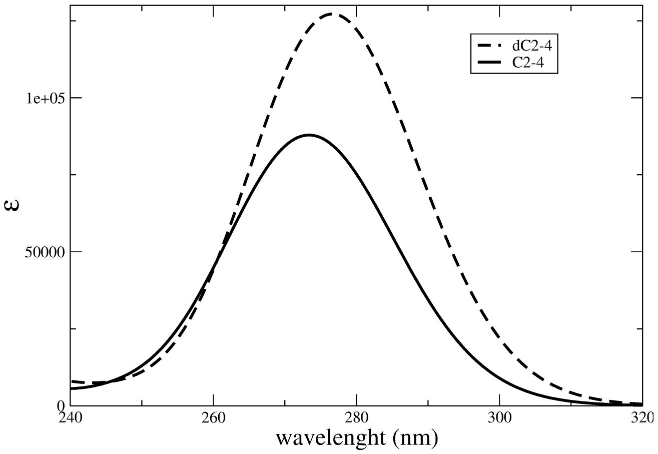

Figure S4: Absorption spectra computed for dC2-4 and C2-4. ϵ in L mol^{-1} cm^{-1}. TD-PCM/M052X/6-31G(d) transition shifted by -0.6 eV and broadened with a Gaussian with hwhm=0.2 eV.

In Figure S5 we report the ECD spectra (unshifted) computed different functionals and basis sets. The computed lineshapes are very similar, indicating that our conclusions are solid with respect to the choice of the functional and of the basis set. From the quantitative point of view, the spectra uniformly red-shift by 0.1$\sim$0.2 eV when the size of the basis set increases and when CAM-B3LYP or ωB97XD functionals are used. On the one hand, these results show that a part of the discrepancy with respect to the experiments is related to the choice of the functional (which we have selected due to its good description of the stacking interactions) and of the basis set. However, it is comforting that the smaller 6-31G(d) basis set, which

enables to perform excited state geometry optimizations and frequency calculations with a feasible computational cost, provides a similar description of the ECD shape and, therefore of the interactions between the excited states, to that obtained with a larger basis set.

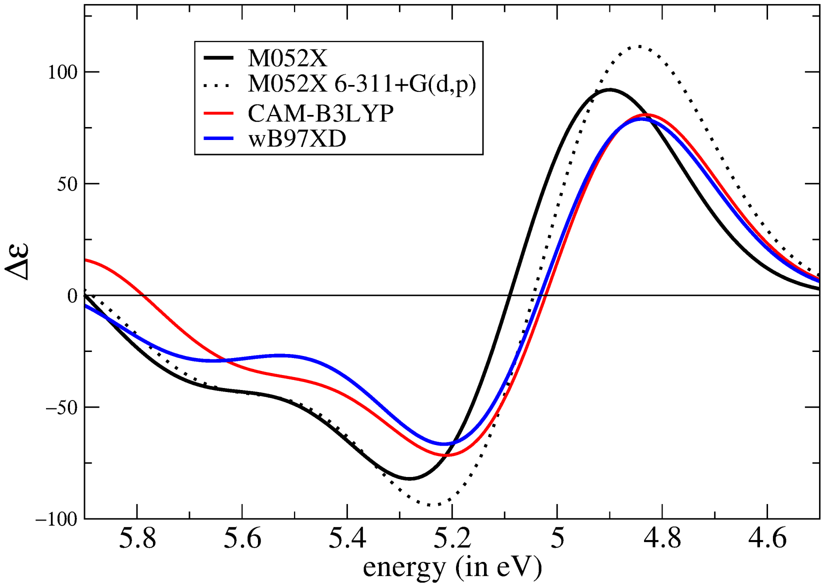

Figure S5: ECD spectra (unshifted) computed for C2-4 using different functionals and basis sets on PCM-M052X/6-31G(d) optimized geometries. TD-PCM/M052X/6-31G(d) transition shifted by -0.6 eV and broadened with a Gaussian with hwhm=0.2 eV. $\Delta\epsilon$ expressed in 10^{-40} esu^2 cm^2.

As anticipated in the main text, the proton 'shared' between the N3 atoms in a C2 pair can 'jump' between the two atoms. As a consequence, for any sequence, many tautomers are possible, differing in the 'location' of the proton. In the presence of the backbone, these tautomers can have different energies. In order to get some insights on the dependence of the spectral shapes on this factor, we have studied four different tautomers of dC2-4, schematically depicted in the top panel of Figure S6. In that scheme, a protonated C is represented by a yellow circle, and the 'unprotonated' one by a green one. For example, in model 1 the two dC_2 dinucleotides are fully protonated and two are not. The relative energy of the possible tautomers, which are very close in energy, will be affected by the length of the chain and by the conformational restrictions due to the presence of the loops, making any analysis of the relative stability of the four species examined not particularly relevant. We decided instead to focus on the spectral properties of the different tautomers. As shown in Figure S6, the four models examined have similar ECD lineshapes. This result indicates

that our conclusions should not depend on the particular dC2-4 tautomer we focused on. On the other hand, the position of the spectra depends on the tautomer considered. This effect contributes to the broadening of the spectral lineshapes and is another effect to be considered when analyzing the effect of the loops on the ECD spectra. It could be indeed possible that the nature and the length of loops could affect the spectra also by affecting the tautomeric equilibria.

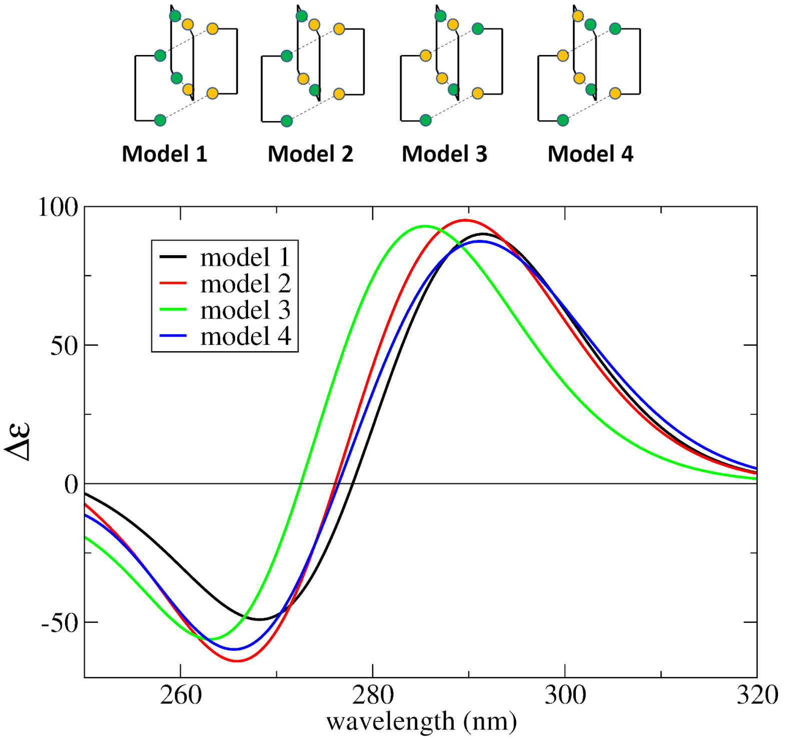

Figure S6: ECD (bottom) spectra computed for dC2-4 species with different hemi-protonation patterns (shown above the spectra, see text fro details). TD-PCM/M052X/6-31G(d) transition shifted by -0.6 eV and broadened with a Gaussian with hwhm=0.2 eV. $\Delta\epsilon$ expressed in 10^{-40} esu^2 cm^2.

In Figure S7 we report the ECD spectra computed for an I-motif (C2-4) and for the two sequences we have used to model 'neutral' conditions, i.e. C2-4-neu and dC$_6$-neu (see Figure S1). Experiments checking the effect of pH on 34-base long cytosine-rich sequence[24, 25] or on (dC)$_{18}$ [26] indicate that for pH$\geq$7.5, where protonation of C is not favored, the typical ECD spectra of I-motifs dramatically change. A positive peak, less intense than that of I-motifs, is observed at $\sim$280 nm, whereas a shallow and not well-structured negative band is present below 260 nm. This spectral shape has been often associated to non-structured

sequences.[27] On the other hand, similar experiments performed on cytosine-rich human telomeric DNA fragments provide a different picture.[28] T he s pectrum m easured a t pH 7.5 exhibits a positive maximum at 275 nm and a negative one, with comparable intensity, around 245 nm. Inspection of Figure S7 provides interesting indications. The ECD spectrum of C2-4-neu exhibits indeed similar features to that usually associated with non-structured conformation. On the other hand, dC_6-neu ECD spectrum is consistent with that measured for the human telomeric sequence at neutral pH, suggesting that it adopts mainly a B-DNA like conformation. It is clear that this can be considered simply a preliminary exploration, which, nonetheless, shows the importance of a careful conformational analysis of the particular C-rich sequence investigated.

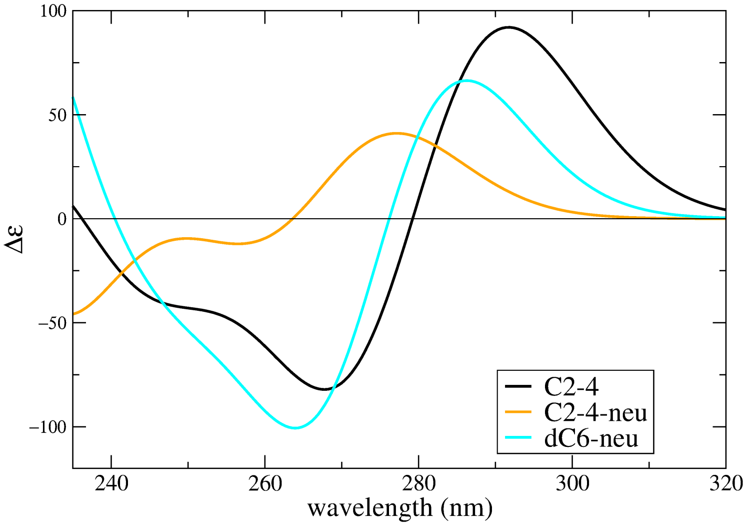

Figure S7: ECD spectra computed for C2-4, C2-4-neu and dC_6-neu. TD-PCM/M052X/6-31G(d) transition shifted by -0.6 eV and broadened with a Gaussian with hwhm=0.2 eV. $\Delta\epsilon$ expressed in 10^{-40} esu^2 cm^2.

S2.4 Assignment of the electronic spectra

S2.4.1 C2 and C2-2 excited states

In Figure S8 we show the NTO's associated to the lowest energy excited states in C2 hemiprotonated pair. As discussed in detail in [12], the lowest energy excited state is a $\pi\pi^*$ HOMO→LUMO transition, localized on the CH^+ base, hereafter CH^+-$\pi_H\pi_L^*$, with bonding/antibonding character with respect to the C5=C6 double bond (Figure S8 top). S_2 corresponds to a similar transition, localized on the C base (hereafter C-$\pi_H\pi_L^*$, see Figure S8 bottom).

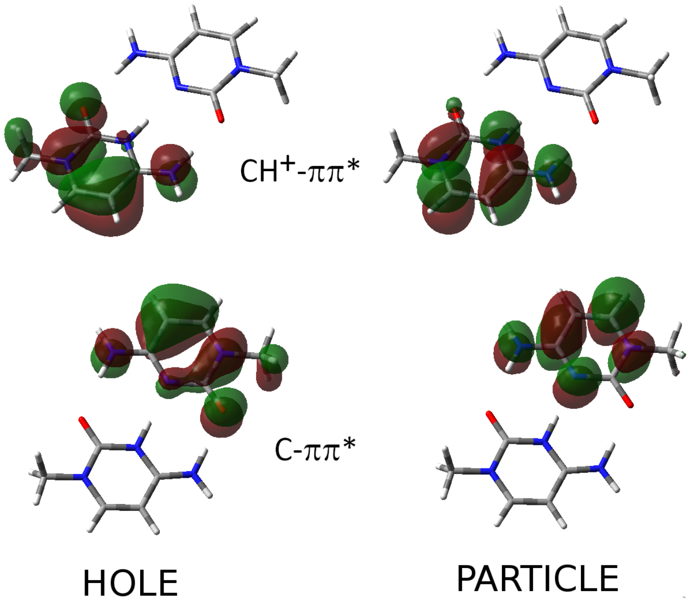

Figure S8: Natural transition orbitals associated to the two lowest energy excited electronic states of C2.

In order to more easily grasp how the formation of I-motif leads to the mixing of these excited states, it is useful to examine C2-2, the minimum meaningful model of I-motifs, since it allows considering both hydrogen bonds and stacking interactions. In C2-2 the two lowest energy excited states essentially derive from the symmetric and asymmetric combination of the CH^+-$\pi\pi^*$ states of the stacked $C^+{\cdot}C$ pairs. The lowest energy one is very weak, and it is red-shifted with respect to the CH^+-$\pi\pi^*$ transition (see Figure S9). In terms of the involved NTOs, for S_1 we find two transitions, with similar weight, where the HOLE are the combinations (symmetric and antisymmetric) of the HOMOs of CH^+ and the Particle of

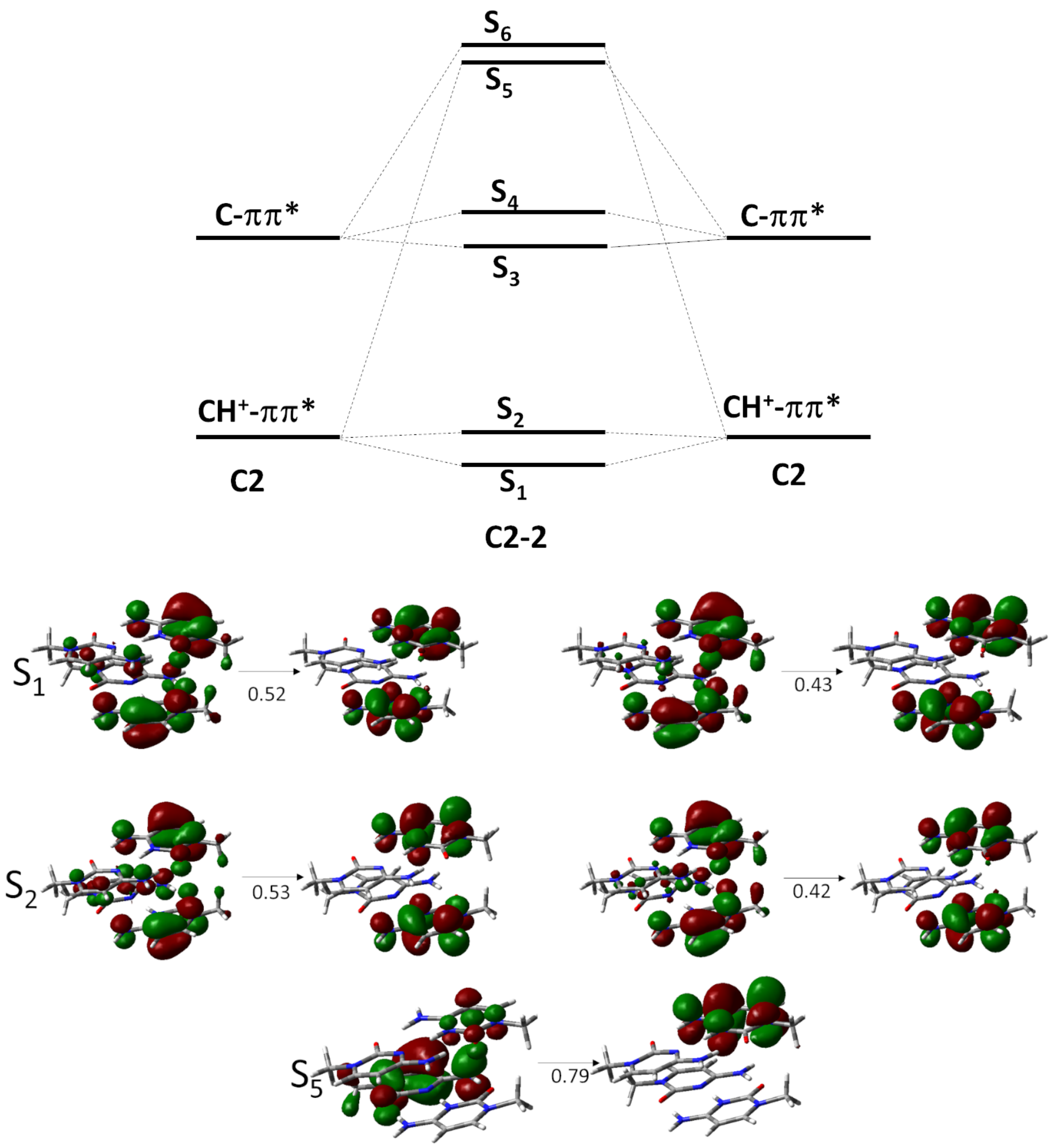

Figure S9: top) Schematic description of the lowest energy excited states of C2-2 in terms of two stacked C2 pairs. bottom) NTOs associated to S_1, S_2 , and S_5. The weight of the most relevant transitions is reported under the arrow.

their LUMOs. The second excited state carries most of the oscillator strength. We then find two excited states deriving from the symmetric/antisymmetric combination of the two C-$\pi\pi^*$ transitions. In this case also, the most intense is that on the blue side, with energy very similar to that of the parent C-$\pi\pi^*$ transitions. The following two excited states, $\sim$0.3 eV on

the blue, can be described as Charge Transfer states, involving the transfer of an electron from a C towards the stacked CH^+. As discussed also in the main text, these assignments provide simply a qualitative description of the dominant character of the transitions. Inspection of Figure S8 shows indeed that S_1 and S_2 have a small C→CH^+ CT character.

S2.4.2 Longer sequences

On the ground of the analysis of C2-2, it is easier to interpret what happens in longer sequences. For C2-3/C2-4, we find three/four exciton states deriving from the mixing of CH^+-$\pi\pi^*$ states, followed by three/four deriving from the mixing of C-$\pi\pi^*$ (see Figure 4 in the main text). Then we find a larger number of excited states with predominant CT character, the most stable being the C→CH^+ ones, involving stacked bases. As discussed in [12] the C→CH^+ ones involving the hydrogen-bonded pair lie $\geq$ 0.6 higher in energy with respect to the bright states.

The contributions of these families of excited states to the ECD spectrum are clearly recognizable in Figure S10, where we report the ECD spectrum of dC2-4, in order to make clearer the assignment of the spectra.

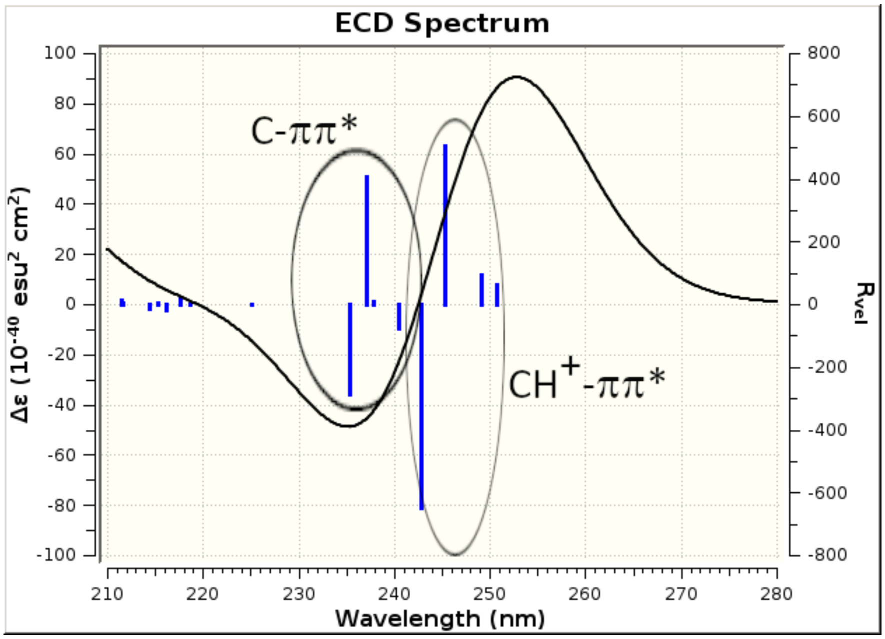

Figure S10: ECD spectra (unshifted) computed for dC2-4. TD-PCM/M052X/6-31G(d) transitions, depicted in blue, broadened with a Gaussian with hwhm=0.2 eV. $\Delta\epsilon$ expressed in 10^{-40} esu^2 cm^2.

There are four contributions around 250 nm associated with the CH^+-$\pi\pi^*$ excitons and four around 240 nm related to the C-$\pi\pi^*$ excitons. In both cases, we have two states with a bidentate strong positive/negative rotatory strength. The states with positive rotatory strength are on the red-wing, explaining the typical ECD signature of I-motifs.

S2.5 Additional details on the excited state minima of C2-4 and dC2-4

In the CH^+-$\pi\pi^*{}^{C}_{T}$-min for C2-4, the C5'=C6' bond distance of the CH^+ base involved in the excitation increases up to 1.416 Å (from the 1.354 Å of the ground electronic state, GS), and on the same time the C4'-N to 1.35 Å (GS 1.312 Å and the C2'=0 to 1.233 Å(in the GS 1.225). At the same time, for the C base involved in the excitation, partially positively charged, the C5C6 bond distance is 1.367 Å (in the GS it is 1.354 Å), and the C4-N distance slightly increases to 1.354 Å , with the amino group getting closer to the C5' atom of the CH^+ base (distance only 2.74 Å) In C-CH^+-CT-min, the two bases involved in the CT transition get much closer with respect to the GS. In particular, we observe the approach of the the C4'-NH_2 moiety of CH^+ to the C5=C6 bond of the C base. The distance between the N'4 amino nitrogen of CH^+ and and the C5 atom of C decreases to 2.56 Å , and that between C4' and C6 to 2.83 Å . At the same time that two base adopt a geometry close to that a C^+ cation and a $CH^{\cdot}$ radical, respectively.

For dC2-4 (as shown in Figure S11), in CH^+-$\pi\pi^*{}^{T}_{C}$-min, the C5'=C6' bond distance of the dCH^+ base involved in the excitation increases up to 1.423 Å (from the 1.354 Å of the GS), and, at the same time, the C4'-N to 1.343 Å (GS 1.312 Å and the C2'=0 to 1.228 Å(in the GS 1.225). For the C base involved in the excitation, which uis partially positively charged, the C5=C6 bond distance is 1.362 Å (in the GS it is 1.354 Å), and the C4-N distance slightly increases to 1.356 Å , with the amino group getting closer to the C5' atom (distance only 2.67 Å) and C6' atom (distance only 2.69 Å) of the CH^+ base. In C-CH^+-CT-min, we observe the approach of the CH^+ and the C bases involved in the CT transition. In particular, the C4'-NH_2 moiety of CH^+ bases gets close to the C5=C6 bond of the C base (bond distance of ∼3 Å see Figure S11), confirming that this minimum could be the

precursor of the photodimer discussed in this study.

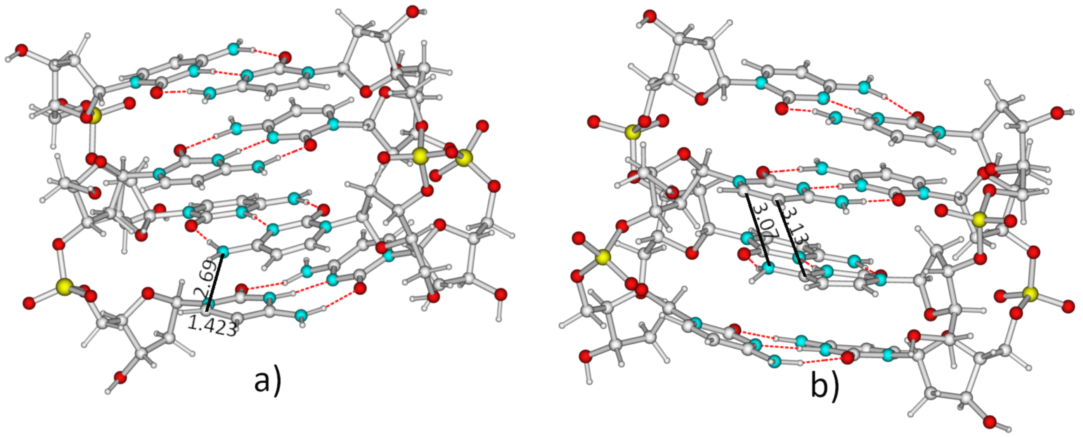

Figure S11: Schematic drawing of the most relevant minima by optimizing the lowest energy excited states of dC2-4. a) CH^{+}-$\pi\pi^{*}_{CT}$-min b) C-CH^{+}-CT-min

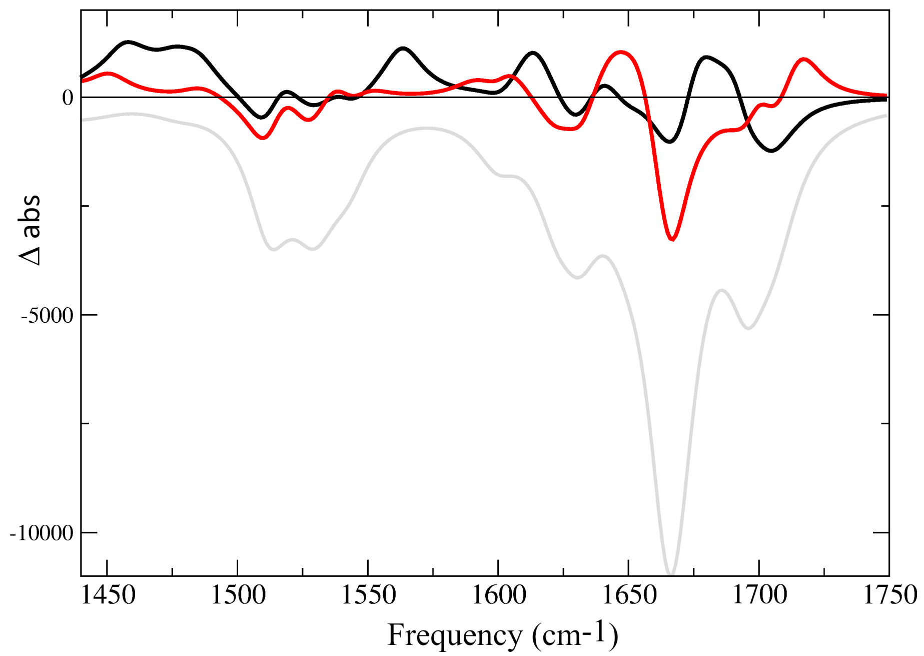

Figure S12: Computed difference IR spectra with respect to the ground electronic state for dC2-4 associated with CH^{+}-$\pi\pi^{*}_{CT}$-min (black line) and C-CH^{+}-CT-min (red line). IR frequencies scaled by 0.955. PCM/TD-M052X/6-31G(d) calculations.

In Figure S12 we report the difference spectra associated to the population of CH^{+}-$\pi\pi^{*}_{CT}$-min (black line) and C-CH^{+}-CT-min (red line) in dC2-4. Considering that the two systems are very different in size and in complexity, they are consistent with those computed for

C2-2 and reported in the main text. Indeed, we observe, fro both excited states, bleaching negative bands associated to the ground state depletion, at ∼1500 cm^{-1}, ∼1620 cm^{-1}, ∼1660 cm^{-1} and 1700 cm^{-1}. We then find positive features below 1500 cm^{-1}, in the region 1550-1600 cm^{-1} and at 1650 cm^{-1}. These features are present for both states, though with different intensity, with trends similar to the discussed in the main text for C 2-2. The most significant differences between the Difference IR spectra computed for the two minima are found in the blue-wing. CH^{+}-$\pi\pi^*_{CT}$-min exhibits a positive feature at 1680 cm^{-1}, whereas C-CH^{+}-CT-min above 1700 cm^{-1}. On the balance, the DIR spectrum of CH^{+}-$\pi\pi^*_{CT}$-min is more consistent with the experimental one, but, considering the approximations of our computational approach, the differences between the DIR computed for the two minima are not large enough to rule out the involvement of C-CH^{+}-CT-min (see the discussion in the main text).

S2.6 Additional details on the dimerization paths

For what concerns the IR spectrum, the dimerization reaction is mirrored by the appearance of positive peaks above 1700 cm^{-1}, at ∼ 1670 cm^{-1}, at ∼ 1650 cm^{-1}, and by a large positive band below 1600 cm^{-1}. At the same time, we observe negative peaks at ∼1690 cm^{-1}, at ∼1660 cm^{-1}, and ∼ 1625 cm^{-1}. These features are nicely consistent with the difference IR spectra measured at low pH after UV irradiation of DNA sequences forming I-motifs (see the black line in Figure S13.[29]

We further investigated the photodimerization reaction, also by using the smaller C2-2 model (see Figure S14). Actually, the rupture of the N4'-C4' bond is moderately exothermic (by ∼0.2 eV for C2-2 and by only ∼0.1 eV for C2-4), producing the species we label as C2-4-dim-break (see Figure S14b). However, it is very likely accompanied by a PT reaction (C2-2-dim-PT in Figure S14c), leading to the formation of a 64-PP dimer involving to C bases hydrogen bonded with two CH^{+} bases.

We have computed the ECD and difference IR spectra associated to these species, which are reported in Figures S13 and S15. Considering the approximations of our computational approach, the computed ECD spectra are consistent with those measured after the production of the photodimer.[29] For what concerns IR spectroscopy, the C2-4-dim-break and C2-4-

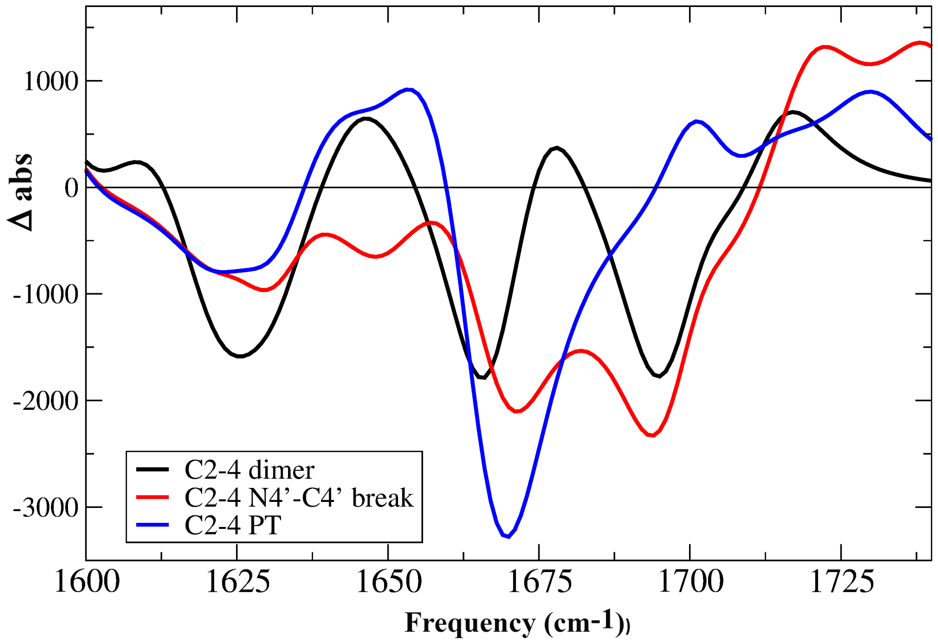

Figure S13: Computed difference IR spectra associated with C2-4-dim, (black line) and C2-4-dim-break (red line), and C2-4-dim-PT (blue line).IR frequencies scaled by 0.955. MC/TD-M052X/6-31G(d) calculations.

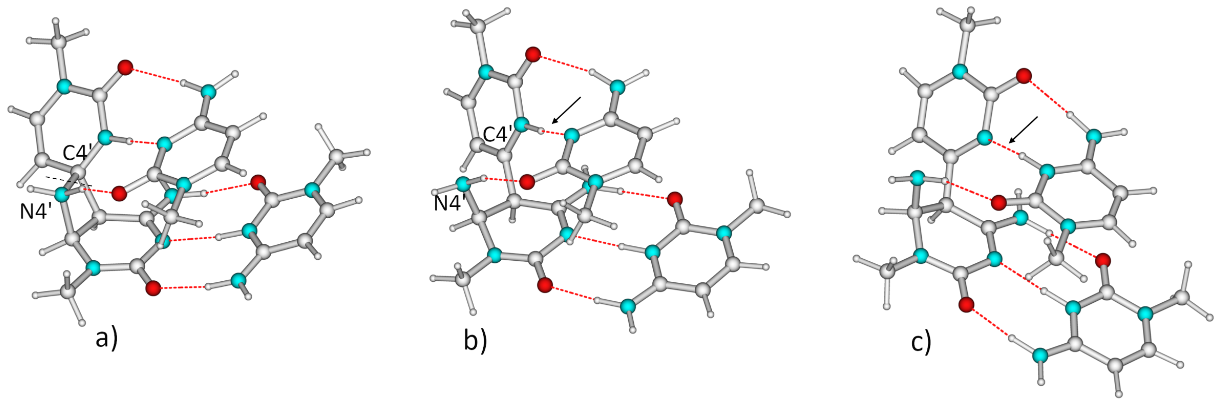

Figure S14: Schematic drawing of different species arising from a) the C2-2-dim photoproduct formed on the surface of C-CH$^+$-CT. Breaking of the C4'-N4' bond can lead to the product shown in b). Then PT transfer in the HB denoted by the arrow can lead to the sepcies shown in c).

dim-PT exhibits DIR spectra less similar to those associated to the photodimer, with respect to that of C2-4-dim.[24] However, we do not consider that photodimerization can affect the stability of the I-motif structure, induce some partial unfolding and/or some structural rearrangements affecting also the the steps not involved in the reaction. All these factors

could affect the DIR spectrum measured after the dimerization. As a consequence, we cannot exclude the formation of C2-4-dim-break and C2-4-dim-PT, simply on the basis of the spectra shown in Figure S13.

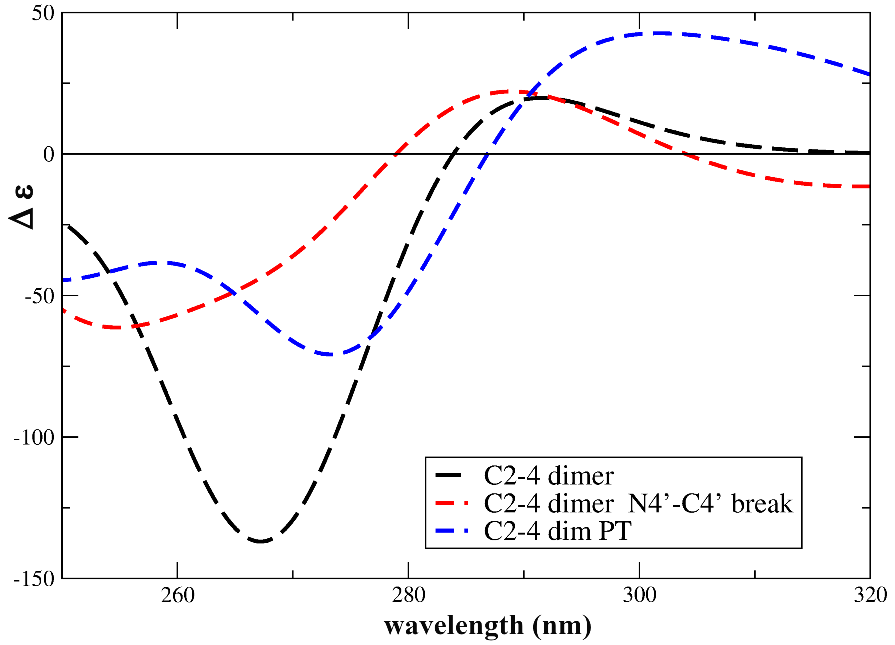

Figure S15: Computed ECD spectra associated with C2-4-dim, (black dashed line) and C2-4-dim-break (red dashed line), and C2-4-dim-PT (blue dashed line). TD-PCM/M052X/6-31G(d) transition shifted by -0.6 eV and broadened with a Gaussian with hwhm=0.2 eV. $\Delta\epsilon$ expressed in 10^{-40} esu^2 cm^2.

References

[1] Dreuw, A.; Head-Gordon, M. Single-Reference ab Initio Methods for the Calculation of Excited States of Large Molecules. *Chem. Rev.* **2005**, *105*, 4009.

[2] Zuluaga, C.; Spata, V.A.; Matsika, S. Benchmarking Quantum Mechanical Methods for the Description of Charge-Transfer States in π-Stacked Nucleobases. *J. Chem. Theory Comp.* **2021**, *17*, 376–387, [https://doi.org/10.1021/acs.jctc.0c00973]. https://doi.org/10.1021/acs.jctc.0c00973.

[3] Zhao, Y.; Truhlar, D.G. Density Functionals with Broad Applicability in Chemistry. *Acc. Chem. Res.* **2008**, *41*, 157–167. https://doi.org/10.1021/ar700111a.

[4] Avila Ferrer, F.J.; Cerezo, J.; Stendardo, E.; Improta, R.; Santoro, F. Insights for an Accurate Comparison of Computational Data to Experimental Absorption and Emission Spectra: Beyond the Vertical Transition Approximation. *J. Chem. Theory Comput.* **2013**, *9*, 2072–2082. https://doi.org/10.1021/ct301107m.

[5] Santoro, F.; Green, J.A.; Martinez-Fernandez, L.; Cerezo, J.; Improta, R. Quantum and semiclassical dynamical studies of nonadiabatic processes in solution: achievements and perspectives. *Phys. Chem. Chem. Phys.* **2021**, *23*, 8181–8199. https://doi.org/10.1039/D0CP05907B.

[6] Phan, A.T.; Guéron, M.; Leroy, J.L. The solution structure and internal motions of a fragment of the cytidine-rich strand of the human telomere11Edited by I. Tinoco. *J. Mol. Biol.* **2000**, *299*, 123–144. https://doi.org/https://doi.org/10.1006/jmbi.2000.3613.

[7] Abou Assi, H.; Garavís, M.; González, C.; Damha, M.J. I-Motif DNA: Structural Features and Significance to Cell Biology. *Nucleic Acids Res.* **2018**, *46*, 8038.

[8] Martínez-Fernández, L.; Pepino, A.J.; Segarra-Martí, J.; Jovaišaitė, J.; Vaya, I.; Nenov, A.; Markovitsi, D.; Gustavsson, T.; Banyasz, A.; Garavelli, M.; et al. Photophysics of Deoxycytidine and 5-Methyldeoxycytidine in Solution: A Comprehensive Picture

by Quantum Mechanical Calculations and Femtosecond Fluorescence Spectroscopy. *J. Am. Chem. Soc.* **2017**, *139*, 7780–7791.

[9] Improta, R.; Santoro, F.; Blancafort, L. Quantum mechanical studies on the photophysics and the photochemistry of nucleic acids and nucleobases. *Chem. Rev.* **2016**, *116*, 3540–3593.

[10] Martínez Fernández, L.; Santoro, F.; Improta, R. Nucleic Acids as a Playground for the Computational Study of the Photophysics and Photochemistry of Multichromophore Assemblies. *Acc. Chem. Res.* **2022**, *55*, 2077–2087, [https://doi.org/10.1021/acs.accounts.2c00256]. https://doi.org/10.1021/acs.accounts.2c00256.

[11] Martínez-Fernández, L.; Esposito, L.; Improta, R. Studying the excited electronic states of guanine rich DNA quadruplexes by quantum mechanical methods: main achievements and perspectives. *Photochem. Photobiol. Sci.* **2020**, *19*, 436–44. https://doi.org/10.1039/D0PP00065E.

[12] Martinez Fernandez, L.; Improta, R. The photophysics of protonated cytidine and hemiprotonated cytidine base pair: a computational study. *Photochem. Photobiol.* **2023**. in press, DOI: 10.1111/php.13832.

[13] Green, J.A.; Asha, H.; Santoro, F.; Improta, R. Excitonic Model for Strongly Coupled Multichromophoric Systems: The Electronic Circular Dichroism Spectra of Guanine Quadruplexes as Test Cases. *J. Chem. Theory Comput.* **2021**, *17*, 405–415. https://doi.org/10.1021/acs.jctc.0c01100.

[14] Asha, H.; Green, J.A.; Esposito, L.; Martinez-Fernandez, L.; Santoro, F.; Improta, R. Effect of the Thermal Fluctuations of the Photophysics of GC and CG DNA Steps: A Computational Dynamical Study. *J. Phys. Chem. B* **2022**, *126*, 10608–10621, [https://doi.org/10.1021/acs.jpcb.2c05688]. https://doi.org/10.1021/acs.jpcb.2c05688.

[15] Asha, H.; Green, J.A.; Esposito, L.; Santoro, F.; Improta, R. Computing the electronic circular dichroism spectrum of DNA quadruple helices of different topology: A critical test for a generalized excitonic model based on a fragment diabatization. *Chirality* **2023**, p. in press, [https://onlinelibrary.wiley.com/doi/pdf/10.1002/chir.23540]. https://doi.org/https://doi.org/10.1002/chir.23540.

[16] Yanai, T.; Tew, D.; Handy, N. A new hybrid exchange−correlation functional using the Coulomb-attenuating method (CAM-B3LYP). *Chem. Phys. Lett.* **2004**, *393*, 51.

[17] Chai, J.D.; Head-Gordon, M. Long-range corrected hybrid density functionals with damped atom–atom dispersion corrections. *Phys. Chem. Chem. Phys.* **2008**, *10*, 6615–6620. https://doi.org/10.1039/B810189B.

[18] Tomasi, J.; Mennucci, B.; Cammi, R. Quantum mechanical continuum solvation models. *Chem. Rev.* **2005**, *105*, 2999–3094.

[19] Green, J.A.; Jouybari, M.Y.; Aranda, D.; Improta, R.; Santoro, F. Nonadiabatic Absorption Spectra and Ultrafast Dynamics of DNA and RNA Photoexcited Nucleobases. *Molecules* **2021**, *26*, 1743. https://doi.org/10.3390/molecules26061743.

[20] Kashinski, D.O.; Chase, G.M.; Nelson, R.G.; Di Nallo, O.E.; Scales, A.N.; VanderLey, D.L.; Byrd, E.F.C. Harmonic Vibrational Frequencies: Approximate Global Scaling Factors for TPSS, M06, and M11 Functional Families Using Several Common Basis Sets. *J. Phys. Chem. A* **2017**, *121*, 2265–2273, [https://doi.org/10.1021/acs.jpca.6b12147]. https://doi.org/10.1021/acs.jpca.6b12147.

[21] Irikura, K.K.; Johnson, R.D.; Kacker, R.N. Uncertainties in Scaling Factors for ab Initio Vibrational Frequencies. *J. Phys. Chem. A* **2005**, *109*, 8430–8437, [https://doi.org/10.1021/jp052793n]. https://doi.org/10.1021/jp052793n.

[22] Capobianco, A.; Landi, A.; Peluso, A. Duplex DNA Retains the Conformational Features of Single Strands: Perspectives from MD Simulations and Quantum

Chemical Computations. *Int. J. Mol. Sci.* **2022**, *23*. https://doi.org/10.3390/ijms232214452.

[23] Keane, P.M.; Wojdyla, M.; Doorley, G.W.; Kelly, J.M.; Parker, A.W.; Clark, I.P.; Greetham, G.M.; Towrie, M.; Magno, L.M.; Quinn, S.J. Long-lived excited states in i-motif DNA studied by picosecond time-resolved IR spectroscopy. *Chem. Commun.* **2014**, *50*, 2990–2992. https://doi.org/10.1039/C3CC46594B.

[24] Benabou, S.; Aviñó, A.; Eritja, R.; González, C.; Gargallo, R. Fundamental aspects of the nucleic acid i-motif structures. *RSC Adv.* **2014**, *4*, 26956–26980.

[25] Benabou, S.; Ferreira, R.; Aviñó, A.; González, C.; Lyonnais, S.; Solà, M.; Eritja, R.; Jaumot, J.; Gargallo, R. Solution equilibria of cytosine- and guanine-rich sequences near the promoter region of the n-myc gene that contain stable hairpins within lateral loops. *Biochim. Biophys. Acta (BBA) - General Subjects* **2014**, *1840*, 41–52. https://doi.org/https://doi.org/10.1016/j.bbagen.2013.08.028.

[26] Cohen, B.; Larson, M.H.; Kohler, B. Ultrafast excited-state dynamics of RNA and DNA C tracts. *Chem. Phys.* **2008**, *350*, 165–174. Femtochemistry and Femtobiology, https://doi.org/https://doi.org/10.1016/j.chemphys.2008.01.050.

[27] Školáková, P.; Renčiuk, D.; Palacký, J.; Krafčík, D.; Dvořáková, Z.; Kejnovská, I.; Bednářová, K.; Vorlíčková, M. Systematic investigation of sequence requirements for DNA i-motif formation. *Nucleic Acids Res.* **2019**, *47*, 2177–2189, [https://academic.oup.com/nar/article-pdf/47/5/2177/28041983/gkz046_supplemental_files.pdf]. https://doi.org/10.1093/nar/gkz046.

[28] Dvořáková, Z.; Renčiuk, D.; Kejnovská, I.; Školáková, P.; Bednářová, K.; Sagi, J.; Vorlíčková, M. i-Motif of cytosine-rich human telomere DNA fragments containing natural base lesions. *Nucleic Acids Res.* **2018**, *46*, 1624–1634, [https://academic.oup.com/nar/article-pdf/46/4/1624/24214813/gky035.pdf]. https://doi.org/10.1093/nar/gky035.

[29] Benabou, S.; Ruckebusch, C.; Sliwa, M.; Aviñó, A.; Eritja, R.; Gargallo, R.; de Juan, A. Study of light-induced formation of photodimers in the i-motif nucleic acid structure by rapid-scan FTIR difference spectroscopy and hybrid hard- and soft-modelling. *Phys. Chem. Chem. Phys.* **2018**, *20*, 19635–19646. https://doi.org/10.1039/C8CP00850G.
